# Gender gap in life expectancy in India and role of age groups: A comparison between before and after male – female life expectancy at birth crossover

**DOI:** 10.1371/journal.pone.0260657

**Published:** 2021-12-02

**Authors:** Girimallika Borah

**Affiliations:** Department of Geography, Cotton University, Guwahati, Assam, India; University of Western Australia, AUSTRALIA

## Abstract

To assess the gender gap in life expectancy at birth in India and its major states as well as the timing of male-female life expectancy at birth crossover. To analyze the age-specific contributions to the changing gender differences before and after the crossover at the national and sub-national levels. We have used sample-survey-based age-specific mortality data available for the periods 1970–2018 to construct abridged life tables. The contribution of different age groups to the gender gap is estimated by using Arriaga’s method of decomposition. During 1981–85 female life expectancy at birth caught up with male life expectancy at birth for India and by 2005 all major states completed the crossover. The male-female crossover in life expectancy at the national level in the early 80s is remarkable in the face of continued female disadvantage from birth till adolescence, even for some richer states. We provide evidence that gender difference in longevity in favour of females is largely a function of adult age groups and younger age groups contribute negatively to the gender gap in life expectancy at birth in most states. Juxtaposing the results from contribution in an absolute number of years and their relative contribution change before and after the crossover, it is established that although the adult and old age groups contribute the highest in the absolute number of years before and after the crossover, the contribution of the reproductive age groups and childhood years in the recent time is most relevant in relative terms.

## 1. Introduction

**Fig 1:**
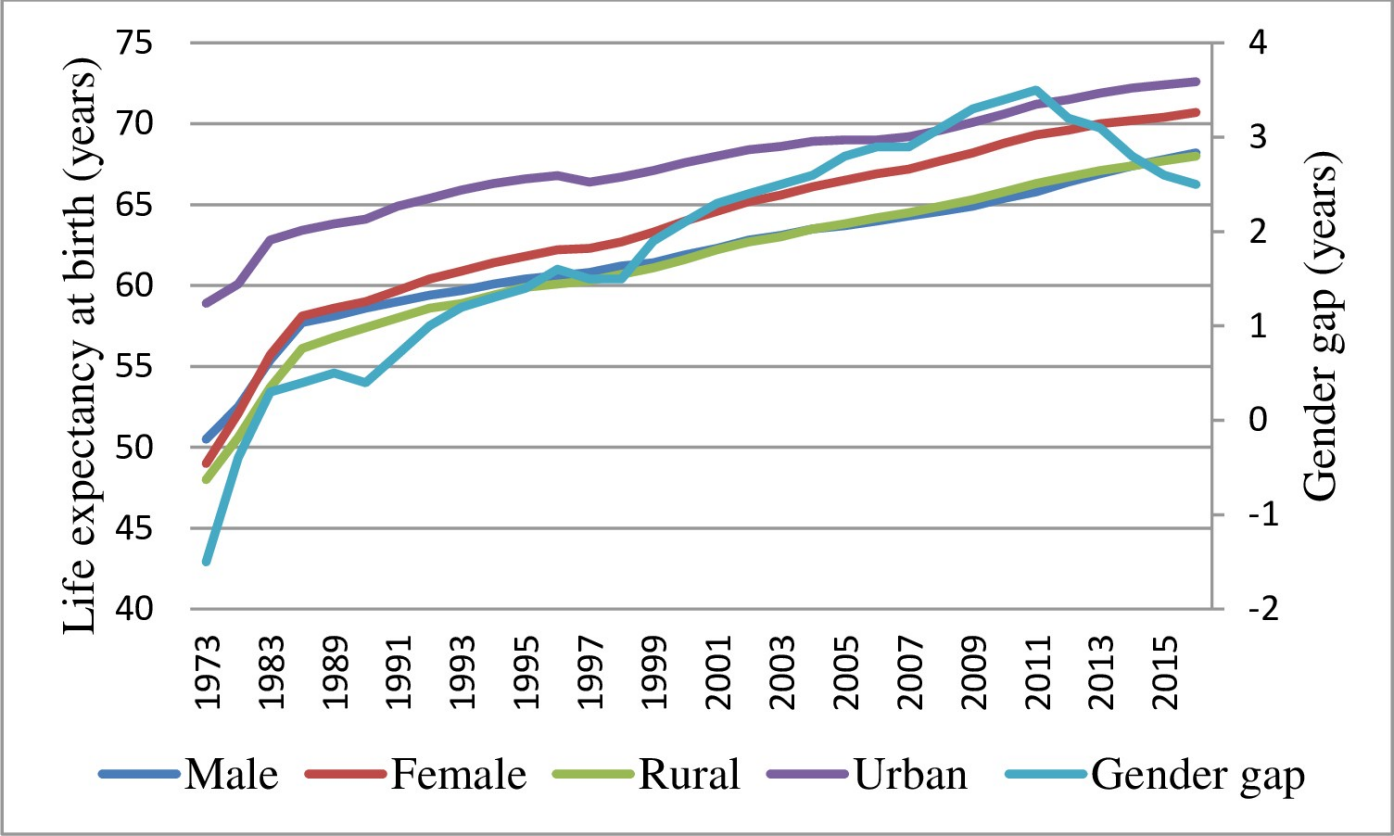
Life expectancy at birth, India, 1970–75 to 2014–18. Source: SRS, 2014–18.

Like many other Asian nations, India began its health transition at a low life expectancy at birth (LEB) of 24.8 years [1]. At the turn of the century, the overall life expectancy in India increased to 62 years. The rate of growth has however slowed down after the 1990s [2]. The average life expectancy of the global population in the year 2019 is 73.3 years [3] and life expectancy in India in 2014–18 is 69.4 years [4]. Not only has India lacked behind international standards, but most of India’s neighbour countries are also doing better than India. Based on World Development Indicators data, it is found that in the year 2019 except for Pakistan and Afghanistan, the rest of South Asian nations have higher life expectancy compared to India [5]. Countries that started their transitions at the same time and at the same level as India have reached higher life expectancy at birth. The reduction and where possible eradication of differentials in mortality has been a primary intention of the World Health Organisation and it is implied in its stated goal of achieving ‘Health for all by the year 2000’ [6]. Life expectancy in India is marked by differences among and between national populations, distinction by sex and place of residence (Fig 1). A better understanding of the age pattern of mortality is required to formulate policy decisions and to follow a target group approach to reduce mortality and improve life expectancy in general.

Another striking distinction of India’s mortality transition is, unlike most of the developed and many developing nations, India continued to have higher male life expectancy when the transition was in process. ‘Crossover’ is a term used in the literature to explain the change in trend. During 1981–85 female LEB had caught up with male LEB at the national level. After the crossover, the gender gap had reached its highest up to 3.5 years in 2009–13 at the national level and has been continuously narrowing ever since. Compared to most developed and many developing nations, the maximum difference in male-female life expectancy that India achieved in 2009–13 is low. Thus, India’s experience is unique in a number of ways. At the sub-national level, the male-female crossover completed by 2003–07 in all the major states for which Sample Registration System (SRS) abridged life tables are available (Table 1). However, a few years later, in 2011–15, another male-female crossover was observed in Bihar when male life expectancy surpassed female life expectancy at birth; the trend of higher male life expectancy over females’ continues in Bihar till the latest available data. Jharkhand is another state from eastern India and showing a similar trend to Bihar. The state was created in 2000 by bifurcating the southern part of Bihar, shows a declining gender gap since 2010–14, since when data is available, and by 2013–17 male LEB surpasses female LEB.

**Table 1:** Year of female crossover male LEB in India and major states, and LEB at the time of crossover.

India and major states	Life expectancy at birth at the time of crossover		Year
	Male	Female	
Andhra Pradesh			Before 1970–1975
Gujarat			Before 1970–1975
Kerala			Before 1970–1975
Maharashtra			Before 1970–1975
West Bengal			Before 1976–1980
Karnataka	56.2	56.6	1978
Rajasthan	51	53	1978
INDIA	55.4	55.7	1983
Himachal Pradesh	58.5	62.9	1983
Jammu and Kashmir	60.2	60.7	1983
Punjab	62.5	53.3	1983
Tamil Nadu	56.5	57.4	1983
Haryana	62.2	62.2	1988
Assam	53.6	54.2	1988
Madhya Pradesh	56.1	57.1	1997
Orissa	57.4	57.5	1997
Uttar Pradesh	60	60.2	2001
Bihar	64.4	64.1	2005
Jharkhand			Before 2010–2014
Chhattisgarh			Before 2010–2014
Uttarakhand			Before 2010–2014

Source: SRS, various years.

Higher life expectancy among females is a general pattern found in the majority of the life tables available across the world and almost universal to the developed countries [7, 8]. Notwithstanding, it is a recent trend in life expectancy in India and a clear north-east and south-west difference is observed among the states regarding the timings of crossover. South and west Indian states passed the crossover earlier than the states located in the north and east India with only two exceptions, West Bengal from the east and Tamil Nadu from the south.

Based on the understanding from the existing literature on the gender gap, India’s experiences are unique in a number of ways. Additionally, at the sub-national level different states are at the various stages of the transition. Age decomposition analysis of the gender gap is done widely because of the availability of the age-specific mortality data and it facilitates comparison among different nations which are at different stages of the transition. To compare attributions of age groups to the gender gap we have performed age-decomposition analysis. In order to understand which age groups contributed, positively or negatively, to the gender gap in LEB before and after the crossover, we have identified three time periods. In this paper, we shall assess the gender gap in life expectancy at the state level. We shall also examine the changing contributions from different age groups to the change in situation from 1970–75 when life expectancy at birth was in favour of males to 1981–85 when females’ advantage over male life expectancy at birth had started and compare contributions from different age groups after the crossover using the time periods 1981–85 to 2014–18.

**Fig 2:**
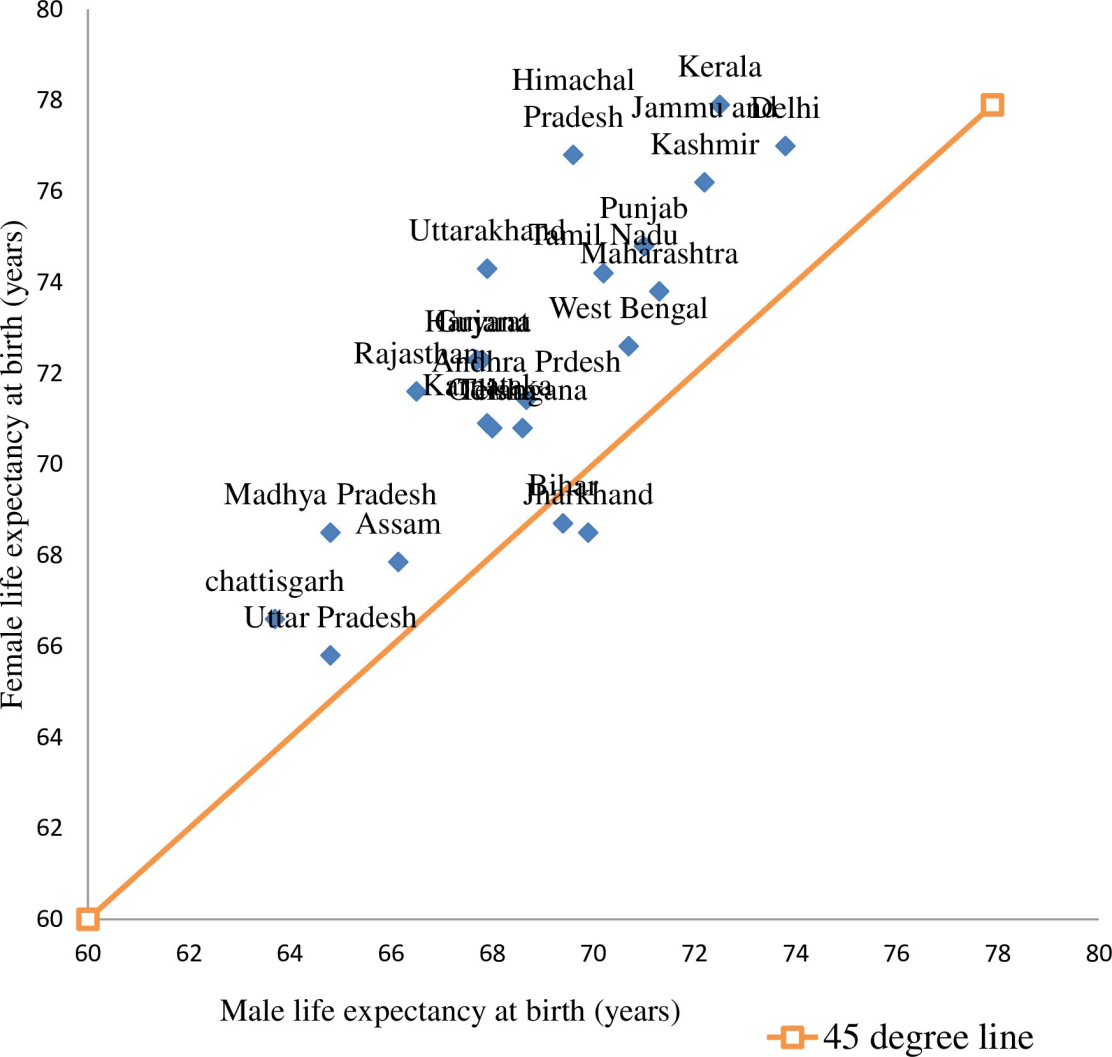
Life expectancy at birth, male and female, major states, 2014–2018. Source: SRS, 2014–2018.

Fig 2 shows the differences in male and female life expectancy at a particular level of life expectancy at birth at the sub-national level during 2014–18. Two states–Bihar and Jharkhand lie below the 45° line. It is very clear that by 2014–18 female life expectancy for all states, except those two, are higher at a given level of male life expectancy. As the gap between the position above the 45° line increases, the gender gap in life expectancy in favour of females increases. Uttar Pradesh, West Bengal, and Assam–from their positions just above the 45° line, it is apparent that sex differentials in life expectancy are very less. The highest gap in LEB in this particular time period is observed for Himachal Pradesh (7.2 years), Uttarakhand (6.4 years), Kerala (5.4 years), and Rajasthan (5.1 years).

## 2. Review of literature

Biologically for many different species, higher mortality among males than females has been observed [5]. There are some interesting findings related to the pattern of sex differentials in LEB among humans. One, higher life expectancy among females is a general pattern found in the majority of the life tables available across the world and almost universal to the developed countries [7, 8]. It is observed in literature from the developed nations that the gender gap in life expectancy at birth in favour of women has been a historical phenomenon. In the history of Europe and North America, women have enjoyed a higher life expectancy over the last 200 years [9]. In Sweden, where life expectancy at birth continued to be the highest until Japan took over, a higher female life expectancy of average 3 years since the 1750s was observed; the increasing trend continued until it started falling in 1978 when the maximum gap between male and female LEB was observed [10]. In New Zealand, the highest gender gap of 6 years was observed in the 1970s, declined afterwards to 4.8 years in 2000–2002 [11]. In Glei and Horiuchi’s work based on 29 high-income countries, a comparison of sex differences in life expectancy across time and space is provided [8]. Based on their analysis it is understood that, in England, Wales, The Netherlands, Iceland, Italy, Finland, Switzerland, and Norway, female life expectancy at birth has been higher than male since the mid-nineteenth century, ever since data is available. In other countries of Europe like Italy, Finland, and Switzerland where data became available since the 1870s, the gender gap was found to be around 2–4 years. In Greece, the gender gap continued to increase till 2009 and subsequently fell [12]. The highest difference between men and women’s LEB was observed in Russia in 1994 at 13.6 years; the trend of the high gender gap continued much later after that. In 2009, the gender gap in LEB was 12.1 years [13].

It is concluded in many literature that the gap increases with the increase in economic development and standard of living [14]. To quote one such assertion, “As society develops, mortality declines and, at the same time, the excess female mortality characteristic of pre-transition societies shifts to higher male mortality” [14]. It is also pointed out that economic development is more beneficial to females since absolute and relative increase of life expectancy at birth coincides with an increase in income [15]. The epidemiological transition theory of Omran points out that the change in society from a subsistence economy based on agriculture to a stage of mass consumption and high spending on public welfare is accompanied by a linear increase in life expectancy at birth and an increased gender gap in favour of females [16]. Developed nations started their mortality transition earlier where the gender gap has increased in favour of females, reaching a peak after which the beginning of the reversal of the trend sets in. The narrowing down of the gap of gender differentials in LEB is pointed out to be the new phase of epidemiological transition. In the 1980s and 90s, sex differences in mortality started declining in many parts, mostly in the developed parts of the world, including The USA, Western Europe, and Australia. There is also evidence from countries of Asia and Africa where a similar trend followed albeit much later. For Example, Korea has achieved the highest gender gap of 8 years in the 1980s; subsequently, the gender gap declined [17]. In urban China, the gender gap reached a peak in 2007 [18]. In Zambia, the gap widened to 4.2 years in 2010 [19]. Japan is an interesting case in this context, where the gender gap had continued to widen and was showing no sign of narrowing down for a long time [20]. Based on a study, it is known that Japan is experiencing the reversal since 2004, after reaching a peak of 7 years in the gender gap in life expectancy [21].

There is a burgeoning literature on various aspects of the gender gap in life expectancy, especially among the developed nations where historical data on LEB is available. One pertinent question is why the gender gap in life expectancy favours women; is the advantage biological or due to behavioural and environmental conditions? Studies have identified the paradox in the gender inequality in the society where men enjoy an advantage in various realms in society but at the same time continue to have lower LEB [22, 23]. Gender inequality is found to be important in the gender gap in life expectancy in the EU [23], men’s mortality risk in the US [22]. Decomposition analysis to identify the age and cause-specific reasons for the gender gap suggests that female survival advantage occurs in the older ages [9, 11, 17, 19, 33].

Two, female disadvantage over male life expectancy in Asia is observed by many scholars. Countries are at various stages of this transition. Interestingly, during the 1950s there were only seven countries in the world where life expectancy at birth for females was lower than that of males and more interestingly six of them were from South Asia [24]. Some of the underlying explanations for female disadvantages in south Asian countries are–high maternal mortality among women in Asia and traditional family and social system where sons are given greater importance and provided with better educational and health facilities than daughters. Environmental factors are also added along with genetic- biological causes [6]. Environmental disadvantages among women in developing cancels out the biological advantage they have over men. Additionally, there is evidence that wars [25] and natural disasters [26] affect women more than men.

Excess female mortality is a South Asian phenomenon where higher female mortality than male counterparts is observed from childhood [27]. Several explanations were given for this distinct pattern of negative differentials in mortality among children. Unequal child care and greater care is given for male children is one explanation for the higher mortality among females during childhood. A study based on Delhi among families who moved originally from Uttar Pradesh and Tamil Nadu corroborates the above finding [28]. In Uttar Pradesh female child mortality was higher than male children but malnutrition is more common among boy children. The reason for higher mortality is not for malnutrition among girl children but lack of medical care given to her. On the contrary, in Tamil Nadu when equal medical treatment is given to both the gender, mortality is higher among boys.

Some improvements in the mortality situation particularly among adult women resulted in higher life expectancy among females in Asia, largely due to improvements in public health services in general and family planning services in particular. Due to a decline in fertility, deaths during pregnancy should decline because fewer women will be exposed to the risk of pregnancy-related deaths. However, in their study among SemaiSenoi, a Malaysian Aboriginal group found a declining maternal rate at the same time when the level of fertility was increasing [14]. They argued it was better health care that had led to the significant decline in maternal mortality.

Different hypotheses are presented regarding mortality differentials with age, one hypothesis “the double jeopardy” and “age- as–a–leveller” is the other [29]. The first hypothesis states that being a woman and being old–both are not the very favourable situation, hence, the health situations and mortality is supposed to increase among the older females. A paper among the elderly found that the negative impact of health is associated with elderly women and increases with age [30].

## 3. Materials and methods

The source of data used is Sample Registration System (SRS). In India, life expectancy at five-year age groups has been estimated by Sample Registration System since 1970–75, the latest available for 2014–18. The Sample Registration System is a large-scale demographic sample survey based on the mechanism of a dual record system with the objective of providing reliable estimates of fertility and mortality indicators. There are several methods of constructing life tables; this is one problem of using SRS-based life tables. There are different ways of converting age-specific mortality rates to life table function *nqx*. One problem in using SRS based abridged life tables is that life tables for the years 1970–75 to 1981–85 are based on Grevelli’s method, but life tables constructed afterwards are constructed using MORTPAK 4- a United Nations’ software package for mortality measurement [31]. To nullify this particular problem we have constructed life tables using MORTPAK 4 taking age-specific death data from SRS, male and female separately, for the entire period. SRS based mortality estimates are available for the major states, i.e. with a population of more than 10 million, are used to construct state-wise life tables.

Though life expectancy at birth is higher for females it does not necessarily mean that mortality among females will be lower in all age groups. The sex ratio of mortality (SRM) is calculated for 21 states using the following formula:

$$\text{SRM}=\frac{nMx}{nFx}$$

Where, nMx is the geometric mean of the 5-year age-specific death rate for the age group (X, X+n) from the male life tables in a particular age group. nFx is the corresponding value for the female age group. The sex ratio of mortality is defined as male upon female mortality, if the value is unitary male and female mortality at that particular age group will be equal. SRM value above 1 means male mortality is higher and vice-versa.

Decomposition techniques are used in formal demography, to understand how a change in mortality in a particular age group has contributed to the change in life expectancy between two time periods or between sexes or between any two subgroups of the population. In this analysis, Arriaga’s method based on discrete data is used. Since certain states at certain times had lower female LEB than male LEB when the percentages are shown it goes very high and seems unreasonable and becomes difficult to interpret. If one age group contributed negatively then the other groups contributed more than the actual difference and the actual age differences although for most of the age groups are very small, makes more sense. Total contribution from a particular age group in years is broken down into direct, indirect and interaction effects. Direct and indirect effects are due to the exclusive change in mortality at that particular age group hence both of them together are called exclusive effects. Mortality differs in all age groups by different magnitudes; the interaction effect is due to the indirect and overall effect. The direct effect on life expectancy is due to a change in life-years in an age group due to the change in mortality in that particular age group. The indirect effect captures the number of years change after the end of that particular age interval. The difference in survivors, after mortality change, will be added or subtracted from years lived as they pass through successive ages, assuming that mortality has not changed and remained at the same level. Both direct and indirect effects are generated because mortality has changed only within the age group under study (it is assumed that mortality has not changed in other ages). This gives us the total and exclusive effect [32].

## 4. Results

### 4.1. Sex ratio of mortality

Our findings are based on the sex ratio of mortality values (male rate per female rate) also called excess male mortality, which records roughly constant female advantage in adult ages commencing at around 20 years. The rising gap in life expectancy should translate to a lower sex ratio of mortality in various age groups in India and its states. Male-female life expectancy crossover in the early 1980s is remarkable because excess female mortality in younger age groups is common in India and its states (S1A–S1D Fig). Below unity SRM values are observed among the younger age groups especially up to age 10 in most states. In the case of Uttar Pradesh, the sex ratios of mortality values are below unity till 25–30 years. The ubiquity of the phenomena of higher female mortality in the younger age groups is observed from a wide range of life expectancy at birth, for example, Kerala at 75.3 years to 66.9 years in Assam. The only exception state where the ratios of mortality are higher than unity among all younger age groups is in Andhra Pradesh from the South.

The sex ratio of mortality exceeds one throughout adult life and reaches a peak at the mid-life. With increasing age, the ratio of mortality increases above unity in most states, in some states the increase is more prominent than the other; for instance, in Himachal Pradesh, between 20 to 60 years, the ratios of male death rates are two to three times higher than female death rates. However there are exceptions, two notable exceptions are Bihar and Jharkhand. In Bihar, all age groups above 50 years have a lower than unity sex ratio of mortality; in Jharkhand, seven 5-year age groups above 30 years have lower than unity sex ratio of mortality, demonstrating excess female mortality starting from age 30 (S1C Fig). In Kerala and Karnataka, the sex mortality ratio increases dramatically in younger ages between the 5–15 years. In fact, in Kerala two peaks are observed, one peak is observed between 5–10 years when male mortality is 2.7 times that of the females. Another peak is observed between 25–45 years. Similarly in Karnataka, excess male mortality is observed between the 5–10 years age group (S1A Fig). Himachal Pradesh from North India, where the gender gap in life expectancy is the highest, sex mortality ratio peaks are observed in the adult ages particularly among three age groups, are 30–35, 40–45 and 50–55 years of age. However, barring a few age groups, most states have roughly constant high SRM values above 1 among adults age commencing at about age 20.

The sex ratio of mortality curve shows a decline in older years meaning a steeper decline in male mortality than that of females. S1E Fig shows the sex ratio of mortality for the three time periods in India. The ratios in the adult ages continue to increase with time. During 1970–75, life expectancy was 49.7 years, men enjoyed lower mortality than women till two-thirds of their lifetime. Life expectancy increased to 55.4 years in 1981–85 but female excess mortality continued in the initial 30–35 years. In 2014–18, LEB increased to 69.4 years and age groups that experiences excess female mortality shrank to the initial10 years.

### 4.2. Contribution of age groups to gender differentials in life expectancy at birth

An earlier study on the temporal increase in temporary life expectancy between ages 0 to 60 estimated a very high contribution from the below five age groups for both sexes, especially during the time period 1970–75 to 1986–90 [2]. Mortality decline among the younger section of the population, however, has proved to be beneficial more for males than for females. The recent change (2014–18) in gender differences in LEB at the national level is largely a function of mortality decline in the 60+ and 45 to 60 years age group; between these ages 45 to 60, two peaks were observed (S2C Fig). Contribution to the gap between male and female LEB rose with age and fell towards the old-olds. Initial age groups up to age 10 negatively contribute to the gender difference in LEB. Earlier, in the previous two time periods (1970–75 and 1981–85), all 5-year age groups below 35 years contributed negatively to the gender gap, favouring men (S2A and S2B Fig). The period of initial disadvantages has shortened for the Indian females now. Levels of infant and child mortality although is decreasing still high in some states. Due to high mortality levels in these age groups coupled with excess female mortality, they contribute significantly but negatively to the absolute gap in LEB. Another inference easily drawn is, for all the three time periods, the direct effect of the change is much lower than the indirect effect of change in mortality. In the figures (S2A–S2C Fig) the curve showing total effect closely corresponds to the curve showing indirect effect. Indirect effects are the result of the number of years added or removed due to mortality change in a particular age group and its resultant change in the number of survivors at the end of the age group.

Age decomposition analysis at the sub-national level for 2014–18 suggests a negative or positive but low contribution from the initial years of life in all states, the disadvantage varies depending on the state. For instance, the initial disadvantages are less pronounced in Karnataka where only the 0–1 year group contributes negatively (S3A Fig), but it continues from birth to age 30 years in Uttar Pradesh (S3B Fig). There are exceptions, one such exception is Himachal Pradesh, where mortality decline among infants contributed nine per cent to the gender gap in LEB, although the following 5-year age groups from 1 to 20 all contribute negatively (S3B Fig). In 2014–18, the age pattern of the contribution to the gender gap in LEB reveals that except for Himachal Pradesh, Uttarakhand, Rajasthan, and Kerala, curves for the rest of the states are much smoother and uniform. In Kerala, Uttarakhand, and Gujarat the peak is seen among the old olds, around 65–70 years of age while in other states the peak is located among the young olds (S3A, S3B and S3D Fig).

We have estimated that barring a few age groups, there are constant high SRM values above 1 in all states in the adult age groups starting at age 20. The high death rates for males in adult age groups between ages 20 to 85+ add 7 and 6 years to the gender gap in LEB in Himachal Pradesh and Uttarakhand respectively (S3B Fig). On the other hand, death rates in all age groups between ages 20 to 85+ shrink the gender gap by 1 and 0.27 years in Jharkhand and Bihar respectively (S3C Fig). Mortality differences in the older age groups over 60 years in India contribute 45 per cent of the 2.5 years gender gap in 2014–18. In an absolute number of years, the contribution of the above 60 is increasing. At the sub-national level, this trend of significantly high contribution from ages above 60 follows, with the notable exception of Maharashtra and Andhra Pradesh where the contribution is less than 30 per cent. Due to the ongoing increase in life expectancy among the older ages, the high contribution and the shifting of peak to the old-old age are happening. The significant contribution from older ages above 60 years is observed in other parts of the world as well, especially after the 1950s [9, 11, 17, 19, 33].

One related question is how much of the increasing gender gap in life expectancy has come from a change in sex differentials in mortality among the three time periods under study? In the LEB trend in India, 1981–85 is the watershed year when female crossover of male LEB happened; subsequently, the gap continued to rise and reached the highest 3.5 years in 2011. Table 2 is the summary finding of differences in contribution from age groups before and following the crossover.

**Table 2:** Age decomposition of female-male difference in life expectancy (the contribution of mortality differences within a given age group to the gender difference in life expectancy at birth and its change between two periods): India.

Age groups	Contribution of a age group in increasing LEB (in absolute years)		Change	Percentage Change
	1970–75 (T1)	1981–85 (T2)	T2 –T1	
00–01	-0.2814	0.002697	0.284099	16.14399
01–05	-1.43151	-1.119867	0.311647	17.70939
05–15	-0.2127	-0.232625	-0.01992	-1.13199
15–45	-0.75273	-0.328406	0.424329	24.11257
45–60	0.664183	0.861075	0.196892	11.18842
60+	0.319415	0.882152	0.562737	31.97762
Total	-1.69476	0.065025	1.759783	100
Age groups	Contribution of a age group in increasing LEB (in absolute years)		Change	Percentage Change
	1981–85 (T2)	2014–18 (T3)	T3 –T2	
00–01	0.002697	-0.18126	-0.18396	-7.57444
01–05	-1.11987	-0.09063	1.029236	42.3785
05–15	-0.23263	-0.01335	0.219275	9.028581
15–45	-0.32841	0.780211	1.108617	45.647
45–60	0.861075	0.874646	0.013571	0.558793
60+	0.882152	1.124085	0.241934	9.961561
Total	0.065025	2.493699	2.428674	100

Source: Author’s calculation based on SRS based abridge life table.

Juxtaposing the results from contribution in the absolute number of years and their relative contribution change over time, it is interesting to note that contributions from the old age groups were distinct before the crossover i.e. from 1971–75 to 1981–85. The shared contribution of 5-year age groups between45-60 and above 60 was positive to the gender difference in life expectancy in favour of females. Although in the absolute number of years the same age groups contribute the highest but relatively, mortality change in the reproductive age group (15–45 years) and in ages 1–5 years are mostly responsible for the increase in the gender gap in favour of females after the crossover. Before the crossover, the relative contribution from different age groups was uniform and except for 5–15 years, all age groups contributed positively towards the gender gap; after the crossover, age groups between 15–45 and 1–5 years explain more than 80 per cent of the gender gap in life expectancy. The 0–1 year age contribute negatively to the gender gap.

## 5. Discussions

Females have had a higher LEB in India since 1983 and by 2005 almost all major states also have higher female LEB. Excess female mortality illustrated by low SRM values is common among ages 0–15 in most states, more prominent in Uttar Pradesh, Assam, Bihar, and Jharkhand in 2014–18. The age decomposition of gender gap results reveal that the initial years of life contribute negatively to the gender gap in LEB. Higher mortality among females between 0–15 years shrinks the gender gap by 0.8 years in Uttar Pradesh and Assam and 0.5 years in Bihar and Jharkhand (S3C Fig). Reducing the overall infant and child mortality and lowering the gender inequality in health among the younger population especially in these four states as well as in Haryana, Punjab, Telangana, and Maharashtra is pertinent. An earlier study on the temporal increase in temporary life expectancy for both genders has confirmed a slowdown in life expectancy gains due to a decline in the pace of under-five mortality reduction [2]. The same study emphasises improvement in neonatal mortality and the nutritional status among mothers and children to further extend the longevity gain.

The mortality disparities in the ages 15 to 45 cause female advantage in LEB, depending on the state, contributes 15 per cent in Himachal Pradesh and Kerala to 50 per cent in Assam. Although at the national level, about one-third of the female survival advantage takes place between ages 15 to 45 in 2014–18 (S2C Fig), there are exceptions among states. We examine how the contribution from different age groups in between the three time periods is changing among the socioeconomically backwards Empowered Action Group (EAG) states and Assam. The eight states from central, north and northeast India viz. Bihar, Chhattisgarh, Jharkhand, Madhya Pradesh, Orissa, Rajasthan, Uttaranchal and Uttar Pradesh are taken for further analysis, although due to data constraints for the initial two time periods, three states viz. Jharkhand, Chhattisgarh, and Uttaranchal are excluded. The total LEB in these six states (including Assam) is low compared to the other states in India. Moreover, evidence from 2014–18 suggests, females of Bihar, Jharkhand, Uttar Pradesh, Assam and West Bengal have lower or slightly higher LEB at a given level of male LEB (Fig 2). Notwithstanding, the total LEB is 71.6 years in West Bengal, therefore, it is not included in the analysis along with the EAG states.

**Fig 3:**
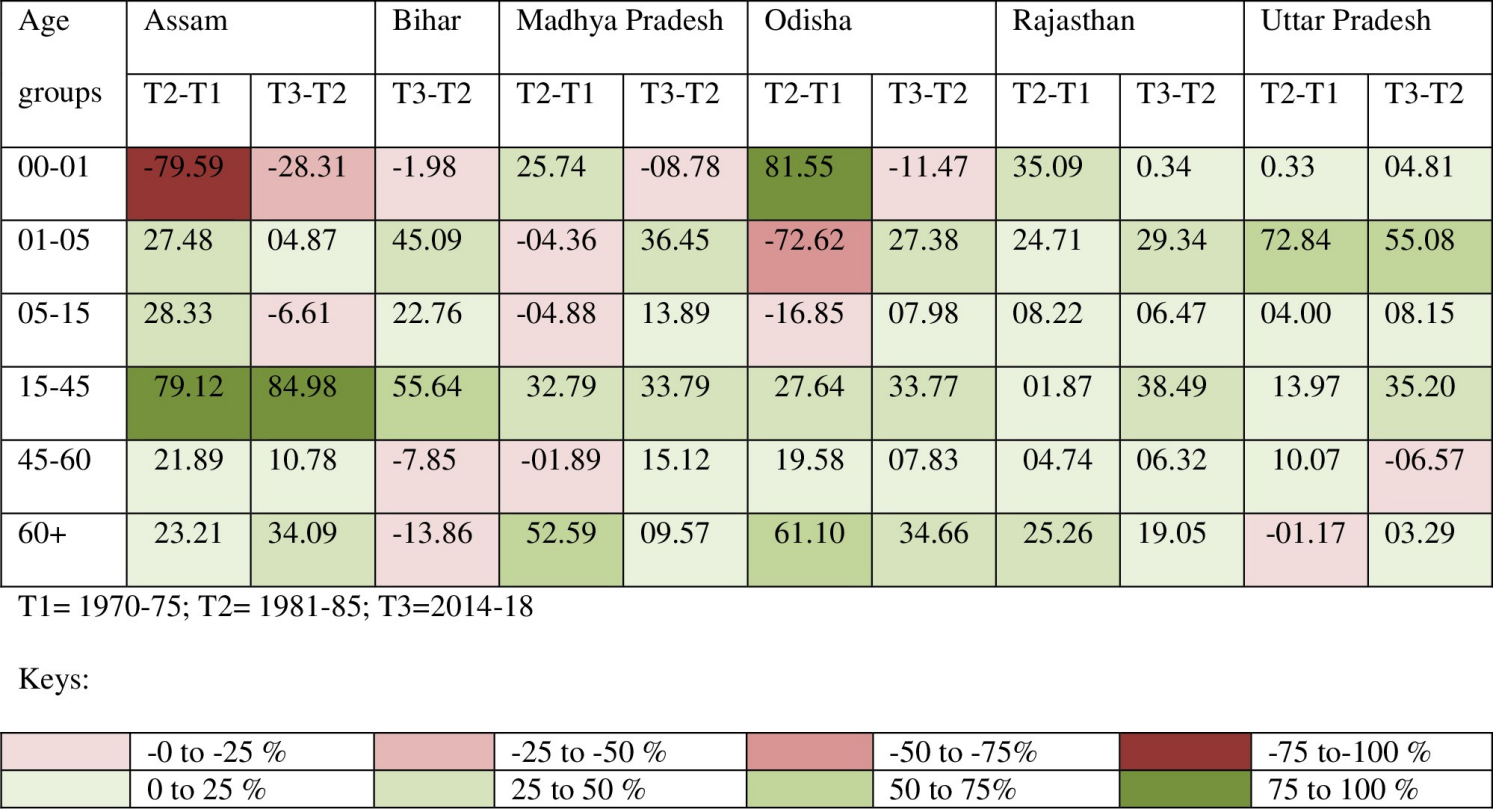
Age decomposition of female-male difference in life expectancy (the relative contribution of mortality differences within a given age group to the gender difference in life expectancy at birth between two periods, in per cent): The Empowered Action Group States and Assam. Source: Author’s calculation based on SRS based abridge life table.

Improvement in survivorship among females in ages 45 to 60 years in Assam and 15 to 45 years in Bihar contribute the highest to the gender gap in 2014-18(S4A and S4B Fig) which may be due to a decline in maternal mortality. Excluding Bihar and Assam, among the remaining four states, an overwhelming contribution to the gender gap now is made by high male mortality after age 60 years (S4C–S4F Fig). Mortality reduction in over 60 years was important even in 1981–85 but 45 to 60 years contributed more. Juxtaposing the absolute and relative contributions to gender gaps in LEB among the five EAG states and Assam, it is observed that, ages 15 to 45 is the only age group that has registered a relative increase in percentages between 1970–75 and 1981–85 to between 1981–85 and 2014–18 for all six states reflecting a growing importance of this age group in gender gap (Fig 3). Female mortality reduction in ages 15 to 45 years contribute an overwhelming percentage to the gender gap in LEB in Assam (85 per cent) and Bihar (55 per cent) after the crossover (Fig 3). This also indicates a high risk of dying in those age groups to make a substantial contribution to the overall increase in life expectancy for females. The risk of maternal mortality in developing countries can be a hundred times higher than the developed countries [34]; these differentials are lower for mortality among children. Based on Sample Registration System’s data for 2016–18, maternal mortality ratios are still very high in Assam, Bihar, Uttar Pradesh, Rajasthan, Madhya Pradesh, Chhattisgarh, and Odisha [35], which suggests that there is room for longevity gain by reducing the maternal mortality. In developed countries up until around 1930s, ages 0 to 40 females were at a disadvantaged position and younger age groups in ages 15 to 40 played only a modest role in the gender gap [33].

## Conclusions

Health transition in India started at a low level of life expectancy at birth and females continued to have lower life expectancy for a long time as the transition was in progress. In the early 80s, female life expectancy exceeded male life expectancy at birth; the gap between male and female subgroups of the population continued to rise till about 2009–13. A reversal of the trend took place from 2009–13 after reaching the highest gap of 3.5 years. Compared to most developed and many developing nations, where females enjoyed a long period of expanding gender gap in their favour, before experiencing a decline, it was a short phase for India. Additionally, the gender gap in life expectancy in absolute years did not reach very high, compared to many other nations. At the sub-national level, states in India have wide differences in the gender gap in life expectancy. The highest gender gap in LEB was observed in Himachal Pradesh with 7.3 years, where the gap is increasing and showing no sign of slowing down. Bihar and Jharkhand are on the other extreme, have lower female LEB and negative differences in the gender gap. A balancing of nature is happening where the advantage made by some states is being reduced by the other states.

This paper tries to analyze the role of different age groups in the gender gap in life expectancy. The contribution of age groups to the gender gap in LEB is measured by Arriaga’s method of decomposition. The result of decomposition analysis for 2014–18 shows positive contribution from ages 15 to 45 and 45 to 60 years, but the mortality after age 60 makes the most significant difference to the gender gap (S2C Fig). The younger ages below 15 contribute negatively to the gender gap in almost all states (S3A–S3D Fig). Thus, the positive contributions made by the older ages are reduced by younger ages. Among the socioeconomically backward EAG state Bihar as well as in Assam, the mortality disparity in the 15–45 and 45–60 age groups contribute an overwhelming percentage to the gender gap (Fig 3). Further decline in maternal mortality in these states can positively impact the female advantage in the gender gap in LEB.

In a recent paper on gender gap in LEB based on evidences from developed nations the growing importance of old ages above 60 is highlighted [33]. Male mortality above 60 years now contribute around 80 per cent in Japan, 70 per cent in Sweden and more than 50 per cent in US. In India, above 60 years contribute in absolute years 0.8 in 1981–85 and 1.2 years in 2014–18 which is about 45 per cent of the gender gap. In 2014–18, although excess male mortality is observed among the adult age groups commencing at around 20 years, the contribution of 15–45 and 45–60 years is a moderate 30–35 per cent each. This is in sharp contrast to the previous two time periods when 15–45 year age group contributed negatively to the gender gap. As the transition is progressing, more and more younger age groups are contributing positively to the gender gap in India. However, Age groups below 15 years continue to contribute negatively. In states with high gender gaps–e.g., Himachal Pradesh in 01–15 years, Kerala in below five years, Uttarakhand in 01–15 years and in state with negative gender gap–e.g., Jharkhand in 0–15 and 45+ age groups -excess deaths among females is narrowing the gender gaps. Reduction in maternal, infant and child deaths as well health equality for both genders especially in those age groups is pertinent. Women although have a biological advantage over males in longevity, lack of care and social norms in India are hindering the longevity potential of their women.

## Supporting information

S1 Fig A. Sex Ratio of mortality, South India, 2014–18. B. Sex Ratio of mortality, North India, 2014–18. C. Sex Ratio of mortality, East India, 2014–18. D. Sex Ratio of mortality, West India, 2014–18. E. Sex Ratio of mortality, India, 1970–75, 1981–85 and 2014–18. Source: SRS and author’s calculation.
(ZIP)

S2 Fig A. Contribution of age groups to the -1.69 years of gender gap in life expectancy at birth between female (48.80) and male (50.49), India, 1970–75. B. Contribution of age groups to the 0.06 years of gender gap in life expectancy at birth between female (55.33) and male (55.26), India, 1981–85. C. Contribution of age group to the 2.49 years of gender gap in life expectancy at birth between female (70.7) and male (68.2), India, 2014–18. Source: SRS and author’s calculation.
(ZIP)

S3 Fig A. Contribution of age groups to gender gap in life expectancy at birth, South India, 2014–18. B. Contribution of age groups to gender gap in life expectancy at birth, North India, 2014–18. C. Contribution of age groups to gender gap in life expectancy at Birth, East India, 2014–18. D. Contribution of age groups to gender gap in life expectancy at Birth, West India, 2014–18. Source: SRS and author’s calculation.
(ZIP)

S4 Fig A. Contribution of age groups to gender gap in 1970–75, 1981–85 and 2014–18, Assam. B. Contribution of age groups to gender gap in 1981–85 and 2014–18, Bihar. C. Contribution of age groups to gender gap in 1970–75, 1981–85 and 2014–18, Madhya Pradesh. D. Contribution of age groups to gender gap in 1970–75, 1981–85 and 2014–18, Odisha. E. Contribution of age groups to gender gap in 1970–75, 1981–85 and 2014–18, Rajasthan. F. Contribution of age groups to gender gap in 1970–75, 1981–85 and 2014–18, Uttar Pradesh. Source: SRS and author’s calculation.
(ZIP)

## References

[1] Riley JC. The timing and pace of health transitions around the world. Popul Dev Rev. 2005 Dec;31(4):741–64. doi: 10.1111/j.1728-4457.2005.00096.x

[2] Saikia N, Jasilionis D, Ram F, Shkolnikov VM. Trends and geographic differentials in mortality under age 60 in India. Popul Stud (Camb). 2011 Mar 1;65(1):73–89. doi: 10.1080/00324728.2010.534642 . 21240833

[3] WHO. Life expectancy and healthy life expectancy data by WHO region [cited Jun 18, 2021]. Available from: https://apps.who.int/gho/data/view.main.SDG2016LEXREGv?lang=en.

[4] Registrar general of India (1970–75, 1981–85, 2014–18). SRS- based abridge life tables of India and the major states. New Delhi: registrar General of India.

[5] WDI, Life Expectancy at Birth, total (Years). Washington, DC: World Bank. World Bank [cited Jun 18, 2021]. Available from: https://data.worldbank.org/indicator/SP.DYN.LE00.IN.

[6] Lopez AD, Ruzicka LT. Sex differentials in mortality: trends, determinants, and consequences: selection of papers presented at the ANU/UN/WHO meeting held in Canberra, Australia, 1–7 December 1981. Canberra: Department of Demography, Australian National University; 1983.

[7] Lee R. The demographic transition: three centuries of fundamental change. J Econ Perspect. 2003 Dec;17(4):167–90. doi: 10.1257/089533003772034943

[8] Glei DA, Horiuchi S. The narrowing sex differential in life expectancy in high-income populations: effects of differences in the age pattern of mortality. Popul Stud (Camb). 2007 Jul 1;61(2):141–59. doi: 10.1080/00324720701331433 . 17558883

[9] Tabutin D, Willems M. Differential mortality by sex from birth to adolescence: the historical experience of the West (1750–1930). In: Too young to die: genes or gender? New York: United Nations; 1998. p. 17–52.

[10] Sundberg L, Agahi N, Fritzell J, Fors S. Why is the gender gap in life expectancy decreasing? The impact of age- and cause-specific mortality in Sweden 1997–2014. Int J Public Health. 2018 Jul;63(6):673–81. doi: 10.1007/s00038-018-1097-3 . 29654335 PMC6015620

[11] Sandiford P. Getting back the missing men of Aotearoa: declining gender inequality in NZ life expectancy. J Prim Health Care. 2009;1(4):270–7. doi: 10.1071/HC09270 . 20690335

[12] Zafeiris KN. Gender differences in life expectancy at birth in Greece 1994–2017. J Popul Res. 2020 Mar;37(1):73–89. doi: 10.1007/s12546-019-09239-4

[13] Cockerham WC. The intersection of life expectancy and gender in a transitional state: the case of Russia. Sociol Health Illn. 2012 Jul;34(6):943–57. doi: 10.1111/j.1467-9566.2011.01454.x . 22497700

[14] Fix AG, FIX AG. Changing sex ratio of mortality in the SemaiSenoi, 1969–1987. Hum Biol. 1991 Apr 1;63(2):211–20. . 2019414

[15] Alachkar A, Serow WJ. The socioeconomic determinants of mortality: an international comparison. Genus. 1988 Jul 1:131–51.

[16] Omran AR. The epidemiologic transition: a theory of the epidemiology of population change. Milbank Mem Fund Q. 1971;49(4):509–38. doi: 10.2307/3349375 . 5155251

[17] Yang S, Khang YH, Chun H, Harper S, Lynch J. The changing gender differences in life expectancy in Korea 1970–2005. SocSci Med. 2012 Oct 1;75(7):1280–7. doi: 10.1016/j.socscimed.2012.04.026 . 22739261

[18] Le Y, Ren J, Shen J, Li T, Zhang CF. The changing gender differences in life expectancy in Chinese cities 2005–2010. PLOS ONE. 2015 Apr 13;10(4):e0123320. doi: 10.1371/journal.pone.0123320 . 25875494 PMC4395256

[19] Chisumpa VH, Odimegwu CO. Decomposition of age- and cause-specific adult mortality contributions to the gender gap in life expectancy from census and survey data in Zambia. SSM Popul Health. 2018 Aug 1;5:218–26. doi: 10.1016/j.ssmph.2018.07.003 . 30094317 PMC6077128

[20] Trovato F. Narrowing sex differential in life expectancy in Canada and Austria: comparative analysis. Vienna Yearbook Popul Res. 2005 Jan 1;1(2005):17–52. doi: 10.1553/populationyearbook2005s17

[21] Liu Y, Arai A, Obayashi Y, Kanda K, Boostrom E, Lee RB, et al. Trends of gender gaps in life expectancy in Japan, 1947–2010: associations with gender mortality ratio and a social development index. GeriatrGerontol Int. 2013 Jul;13(3):792–7. doi: 10.1111/ggi.12001 . 23216600

[22] Kavanagh SA, Shelley JM, Stevenson C. Does gender inequity increase men’s mortality risk in the United States? A multilevel analysis of data from the National Longitudinal Mortality Study. SSM Popul Health. 2017 Dec 1;3:358–65. doi: 10.1016/j.ssmph.2017.03.003 . 29349229 PMC5769061

[23] Kolip P, Lange C. Gender inequality and the gender gap in life expectancy in the European Union. Eur J Public Health. 2018 Oct 1;28(5):869–72. doi: 10.1093/eurpub/cky076 . 29767703

[24] Karkal M. Differentials in mortality by sex. Economic&Political Weekly. 1987 Aug 8:1343–7.

[25] Plümper T, Neumayer E. The unequal burden of war: the effect of armed conflict on the gender gap in life expectancy. Int Organ. 2006 Jul;60(3):723–54. doi: 10.1017/S0020818306060231

[26] Neumayer E, Plümper T. The gendered nature of natural disasters: the impact of catastrophic events on the gender gap in life expectancy, 1981–2002. Ann Assoc Am Geogr. 2007 Sep 1;97(3):551–66. doi: 10.1111/j.1467-8306.2007.00563.x

[27] Pebley AR, Amin S. The impact of a public-health intervention on sex differentials in childhood mortality in rural Punjab, India. Health Transit Rev. 1991 Oct 1;1(2):143–69. . 10148659

[28] Basu AM, Basu K. Women’s economic roles and child survival: the case of India. Health Transit Rev. 1991 Apr 1;1(1):83–103. . 10148805

[29] Knodel J, Ofstedal MB. Gender and aging in the developing world: where are the men? Popul Dev Rev. 2003 Dec;29(4):677–98. doi: 10.1111/j.1728-4457.2003.00677.x

[30] Alam M, Karan A. Elderly health in India: Dimension, Differentials and Determinants. BKPAI Working Paper No. 3 [retrieved Jun 29, 2021 from]. Available from: https://www.researchgate.net/profile/Anup-Karan/publication/275026837_Health_status_of_elderly_in_India_trends_and_differentials/links/608551612fb9097c0c095d56/Health-status-of-elderly-in-India-trends-and-differentials.pdf. New Delhi: United Nations Population Fund (united nations population fund); 2011.

[31] RanjanChaurasia AR. Mortality transition in India 1970–2005. Asian Popul Stud. 2010;6(1):47–68. doi: 10.1080/17441731003603421

[32] Arriaga EE. Measuring and explaining the change in life expectancies. Demography. 1984 Feb;21(1):83–96. doi: 10.2307/2061029 . 6714492

[33] Zarulli V, Kashnitsky I, Vaupel JW. Death rates at specific life stages mold the sex gap in life expectancy. Proc Natl AcadSci U S A. 2021 May 18;118(20). doi: 10.1073/pnas.2010588118 . 33972417 PMC8157960

[34] Rosenfield A. Maternal mortality in developing countries: an ongoing but neglected’epidemic. JAMA. 1989 Jul 21;262(3):376–9 . 2661871

[35] Registrar general of India 2020. Special Bulletin on Maternal Mortality in India. New Delhi: Government of India; 2016–18.

