# Supplementary figures and images for: Gender gap in life expectancy in India and role of age groups: A comparison between before and after male – female life expectancy at birth crossover
Source: PLoS One. 2021 Dec 2;16(12):e0260657. doi: 10.1371/journal.pone.0260657 (PMC8638908; doi:10.1371/journal.pone.0260657)

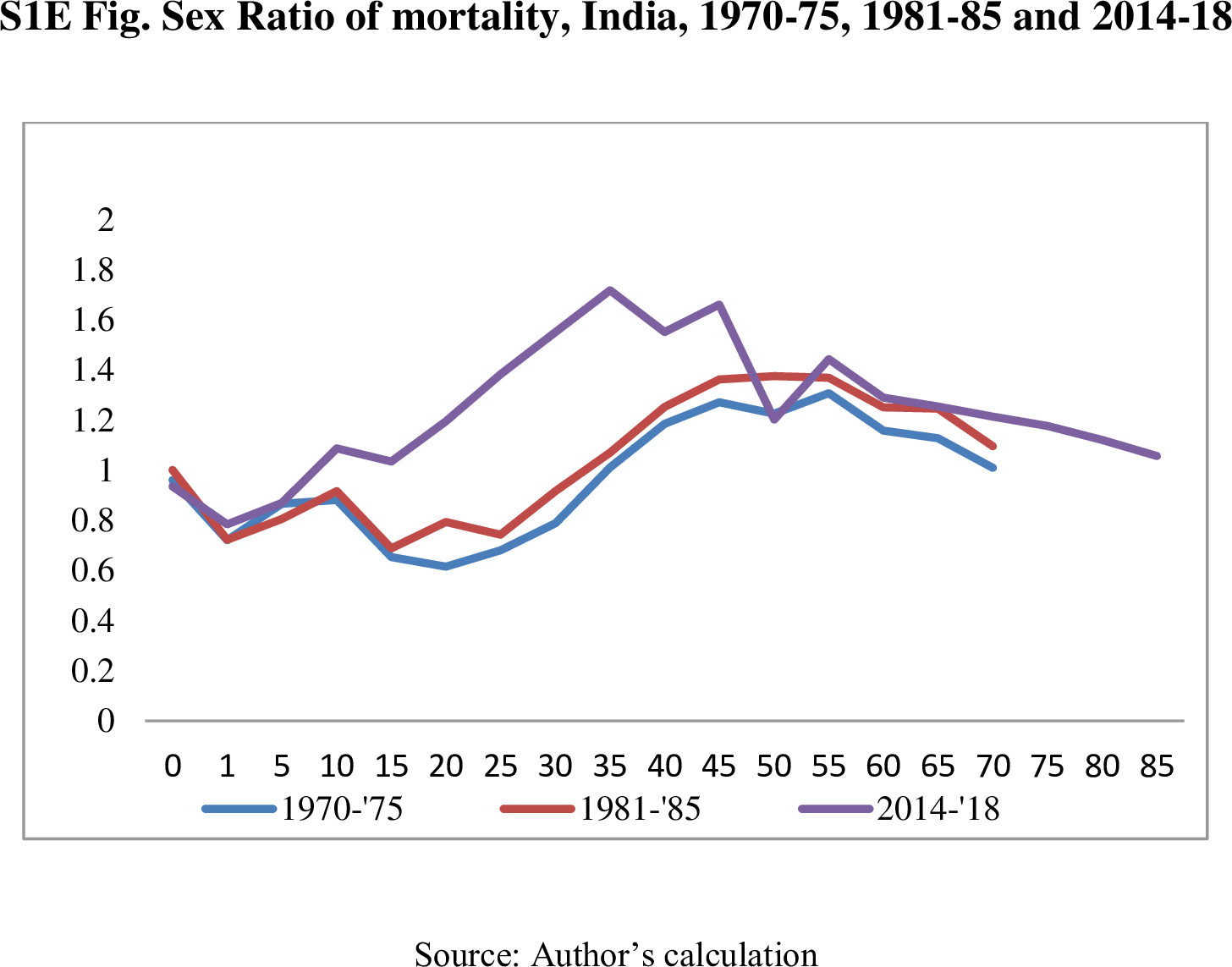

Supplement: S1 Fig — A. Sex Ratio of mortality, South India, 2014–18. B. Sex Ratio of mortality, North India, 2014–18. C. Sex Ratio of mortality, East India, 2014–18. D. Sex Ratio of mortality, West India, 2014–18. E. Sex Ratio of mortality, India, 1970–75, 1981–85 and 2014–18. Source: SRS and author’s calculation. (ZIP) [file pone.0260657.s001.zip › S1E_Fig.tif]

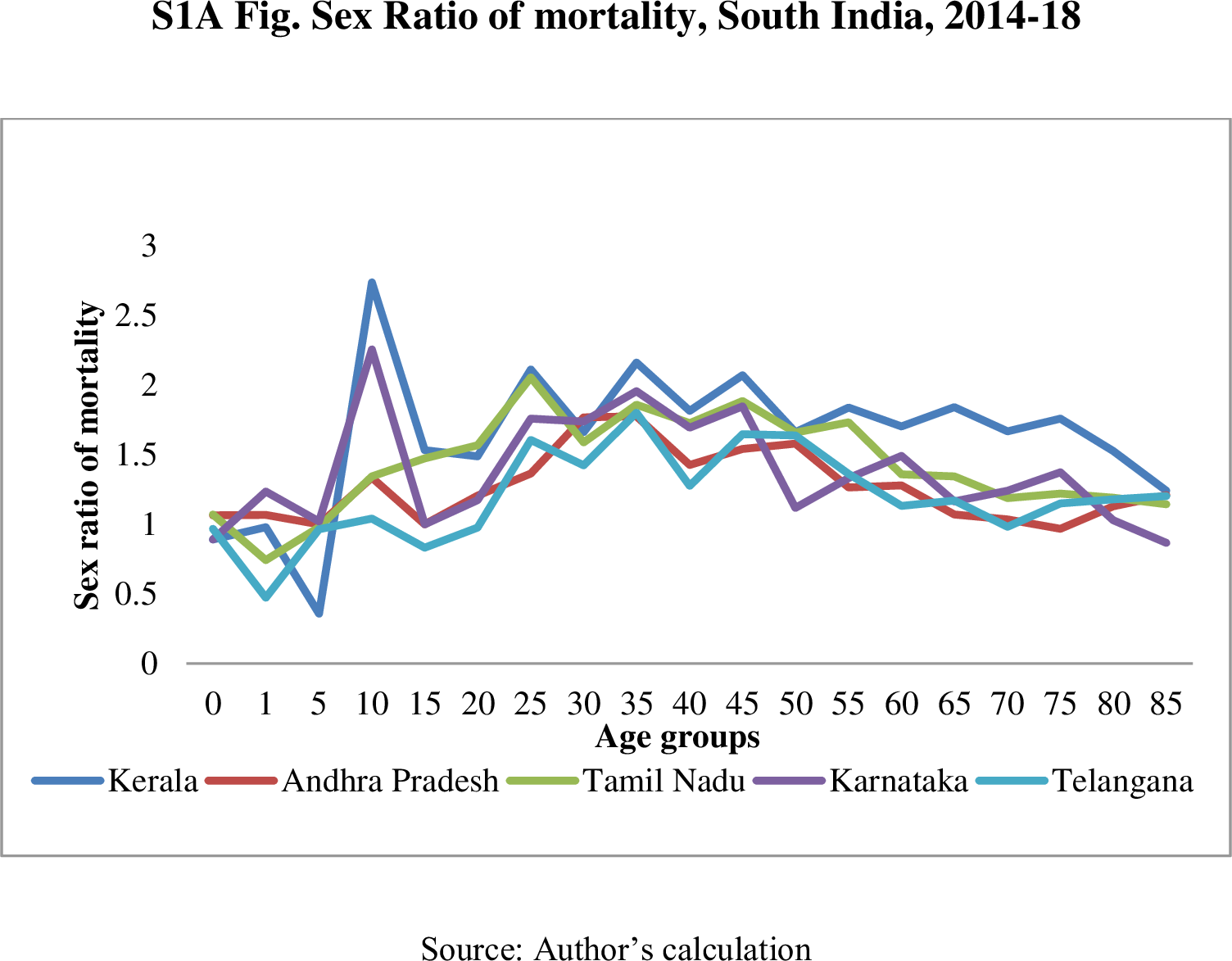

Supplement: S1 Fig — A. Sex Ratio of mortality, South India, 2014–18. B. Sex Ratio of mortality, North India, 2014–18. C. Sex Ratio of mortality, East India, 2014–18. D. Sex Ratio of mortality, West India, 2014–18. E. Sex Ratio of mortality, India, 1970–75, 1981–85 and 2014–18. Source: SRS and author’s calculation. (ZIP) [file pone.0260657.s001.zip › S1A_Fig.tif]

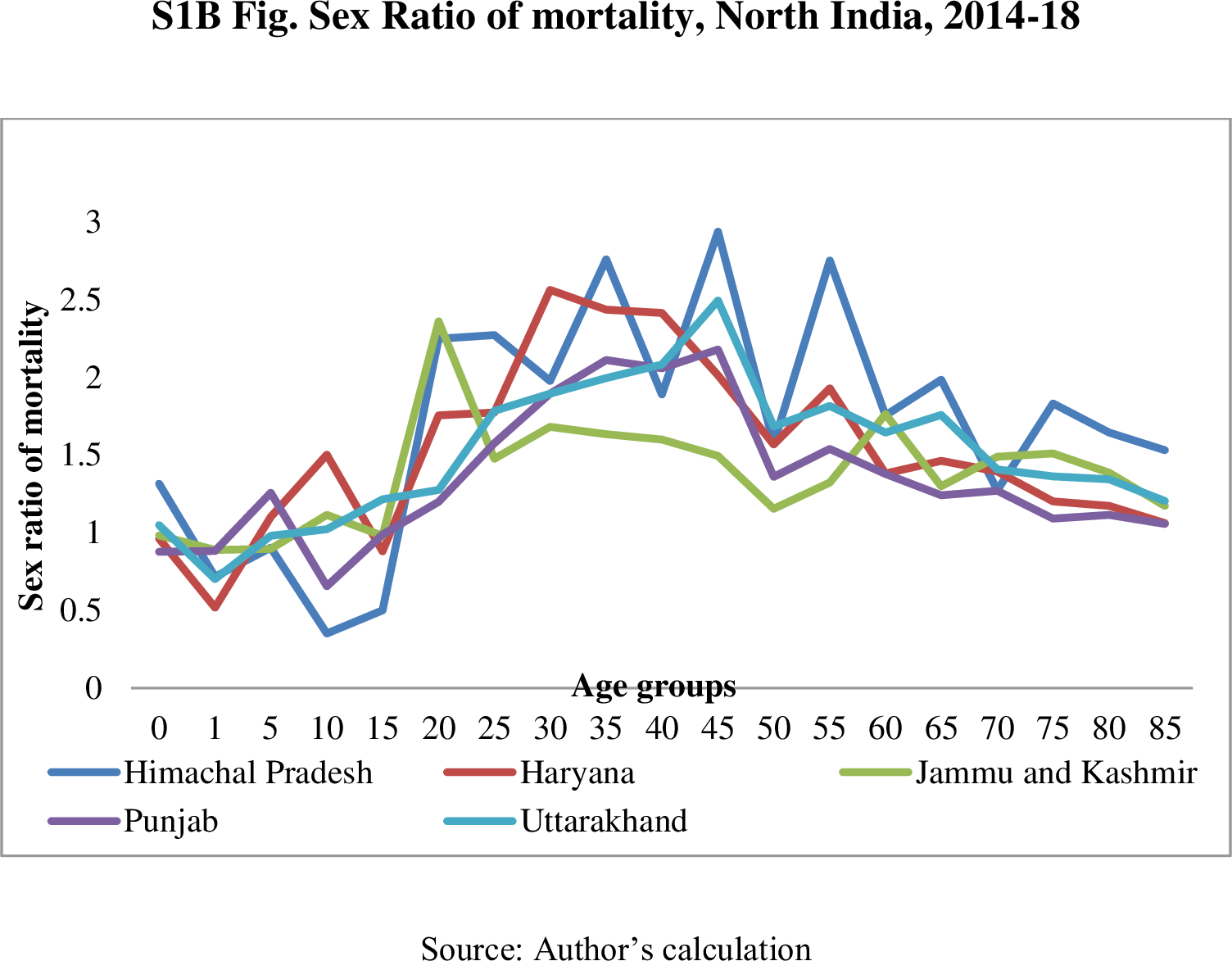

Supplement: S1 Fig — A. Sex Ratio of mortality, South India, 2014–18. B. Sex Ratio of mortality, North India, 2014–18. C. Sex Ratio of mortality, East India, 2014–18. D. Sex Ratio of mortality, West India, 2014–18. E. Sex Ratio of mortality, India, 1970–75, 1981–85 and 2014–18. Source: SRS and author’s calculation. (ZIP) [file pone.0260657.s001.zip › S1B_Fig.tif]

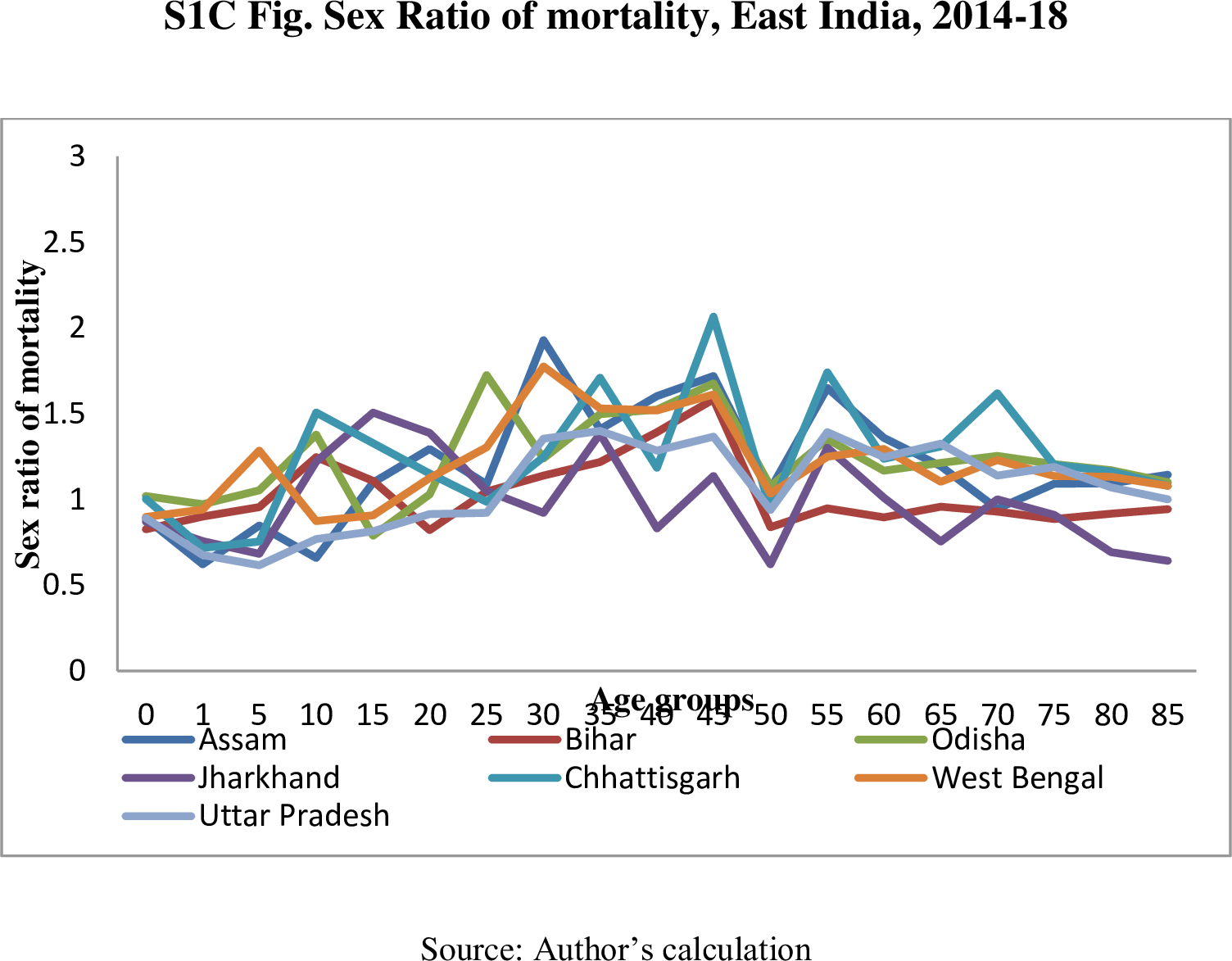

Supplement: S1 Fig — A. Sex Ratio of mortality, South India, 2014–18. B. Sex Ratio of mortality, North India, 2014–18. C. Sex Ratio of mortality, East India, 2014–18. D. Sex Ratio of mortality, West India, 2014–18. E. Sex Ratio of mortality, India, 1970–75, 1981–85 and 2014–18. Source: SRS and author’s calculation. (ZIP) [file pone.0260657.s001.zip › S1C_Fig.tif]

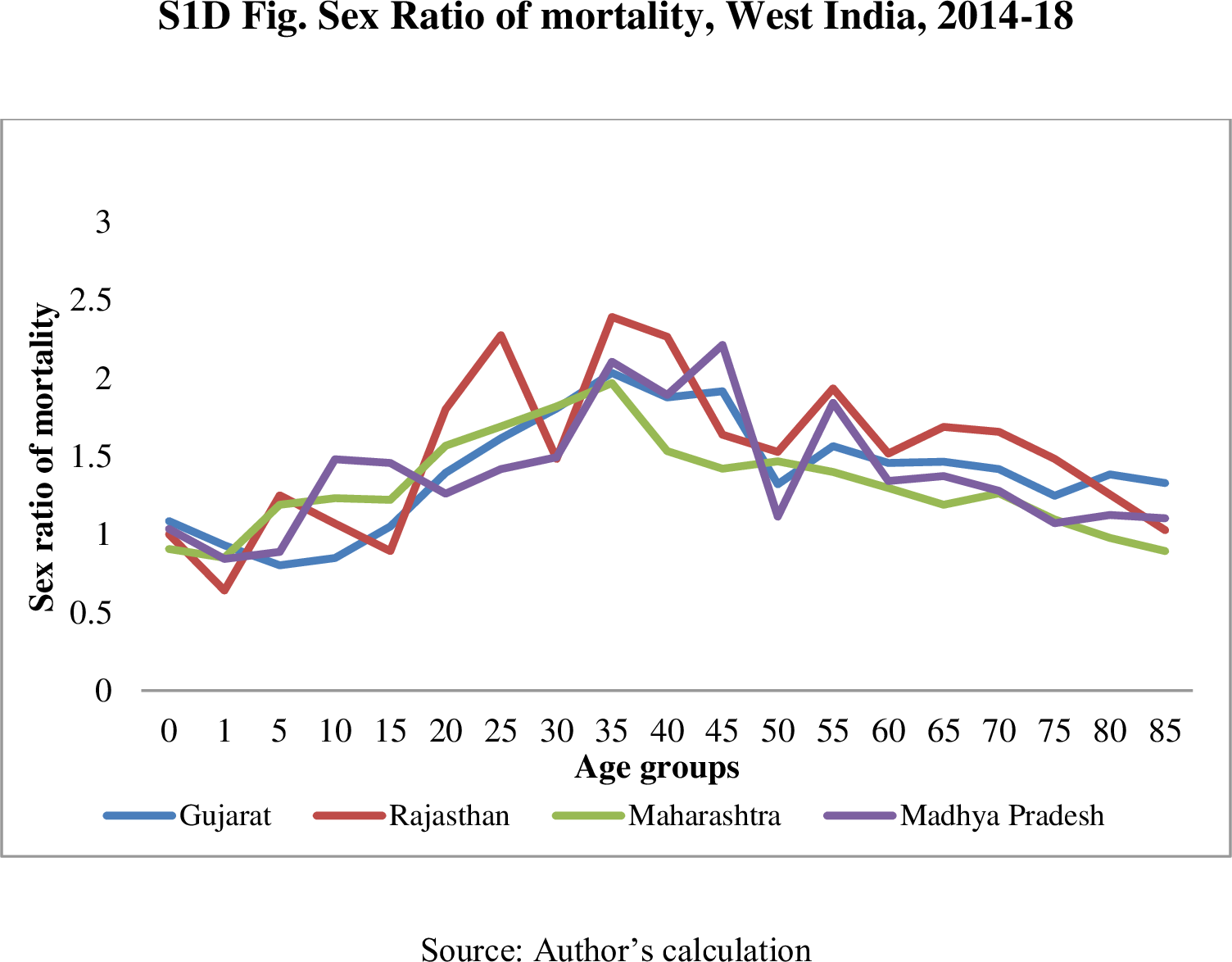

Supplement: S1 Fig — A. Sex Ratio of mortality, South India, 2014–18. B. Sex Ratio of mortality, North India, 2014–18. C. Sex Ratio of mortality, East India, 2014–18. D. Sex Ratio of mortality, West India, 2014–18. E. Sex Ratio of mortality, India, 1970–75, 1981–85 and 2014–18. Source: SRS and author’s calculation. (ZIP) [file pone.0260657.s001.zip › S1D_Fig.tif]

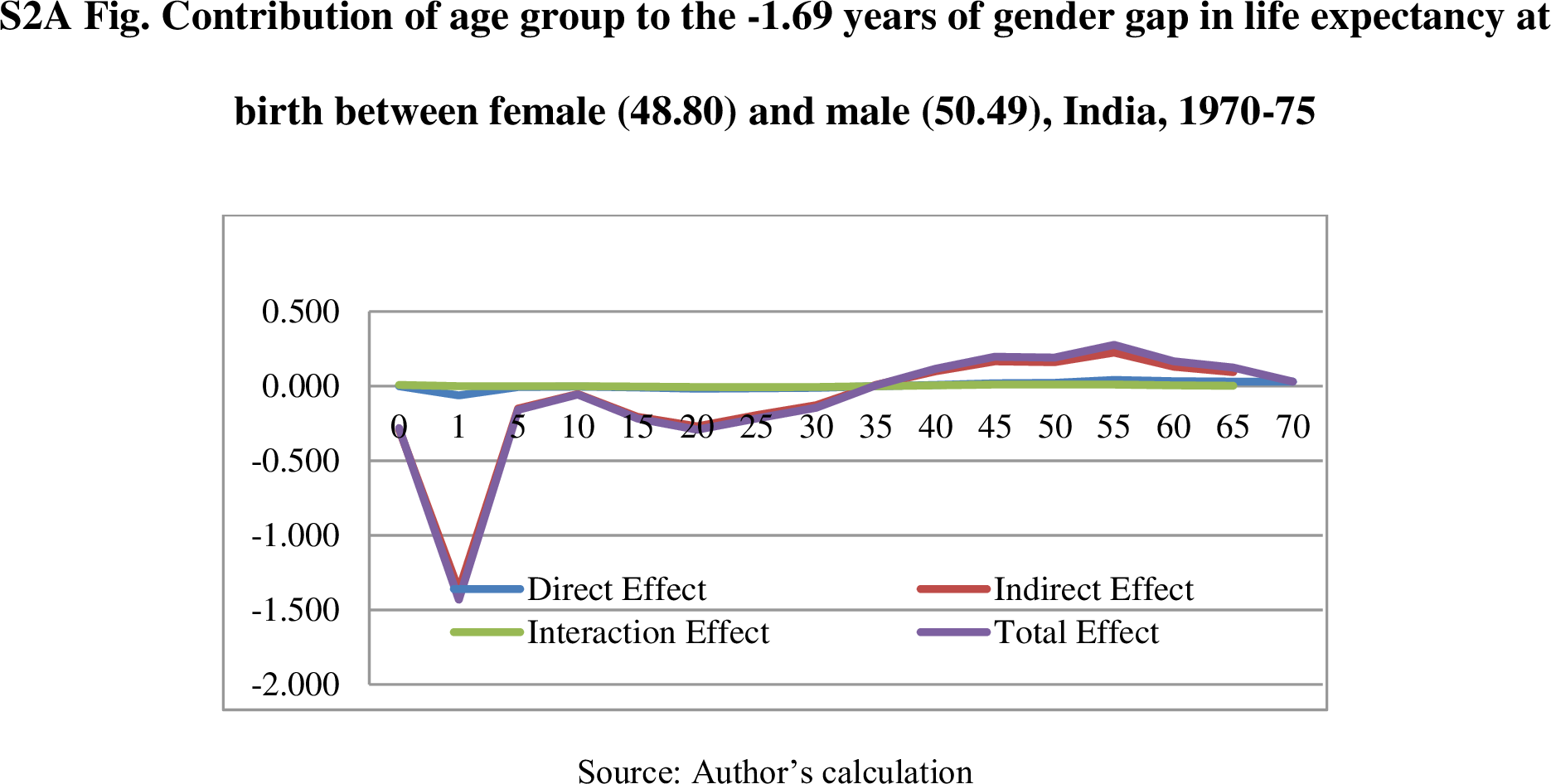

Supplement: S2 Fig — A. Contribution of age groups to the -1.69 years of gender gap in life expectancy at birth between female (48.80) and male (50.49), India, 1970–75. B. Contribution of age groups to the 0.06 years of gender gap in life expectancy at birth between female (55.33) and male (55.26), India, 1981–85. C. Contribution of age group to the 2.49 years of gender gap in life expectancy at birth between female (70.7) and male (68.2), India, 2014–18. Source: SRS and author’s calculation. (ZIP) [file pone.0260657.s002.zip › S2A_Fig.tif]

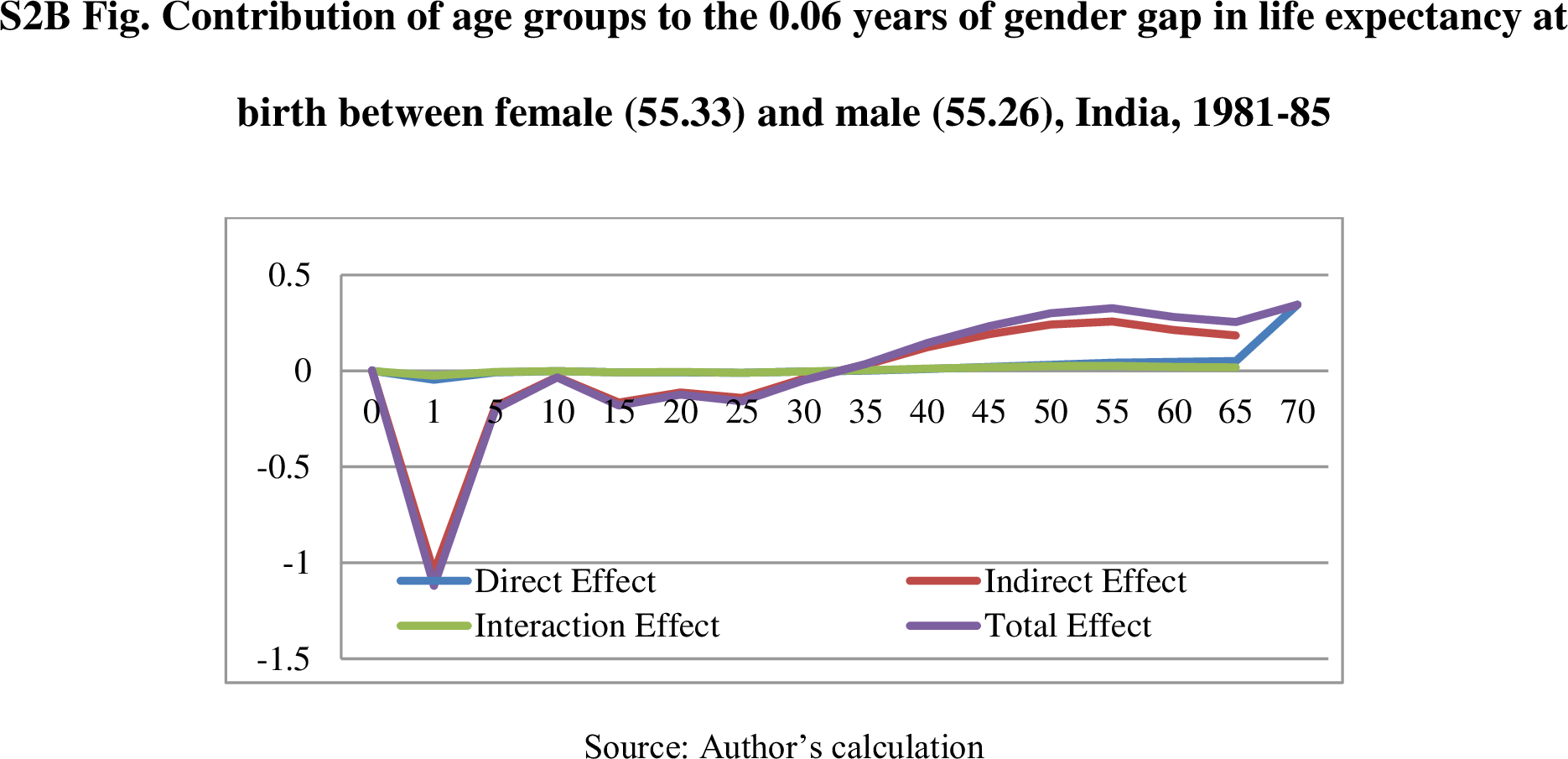

Supplement: S2 Fig — A. Contribution of age groups to the -1.69 years of gender gap in life expectancy at birth between female (48.80) and male (50.49), India, 1970–75. B. Contribution of age groups to the 0.06 years of gender gap in life expectancy at birth between female (55.33) and male (55.26), India, 1981–85. C. Contribution of age group to the 2.49 years of gender gap in life expectancy at birth between female (70.7) and male (68.2), India, 2014–18. Source: SRS and author’s calculation. (ZIP) [file pone.0260657.s002.zip › S2B_Fig.tif]

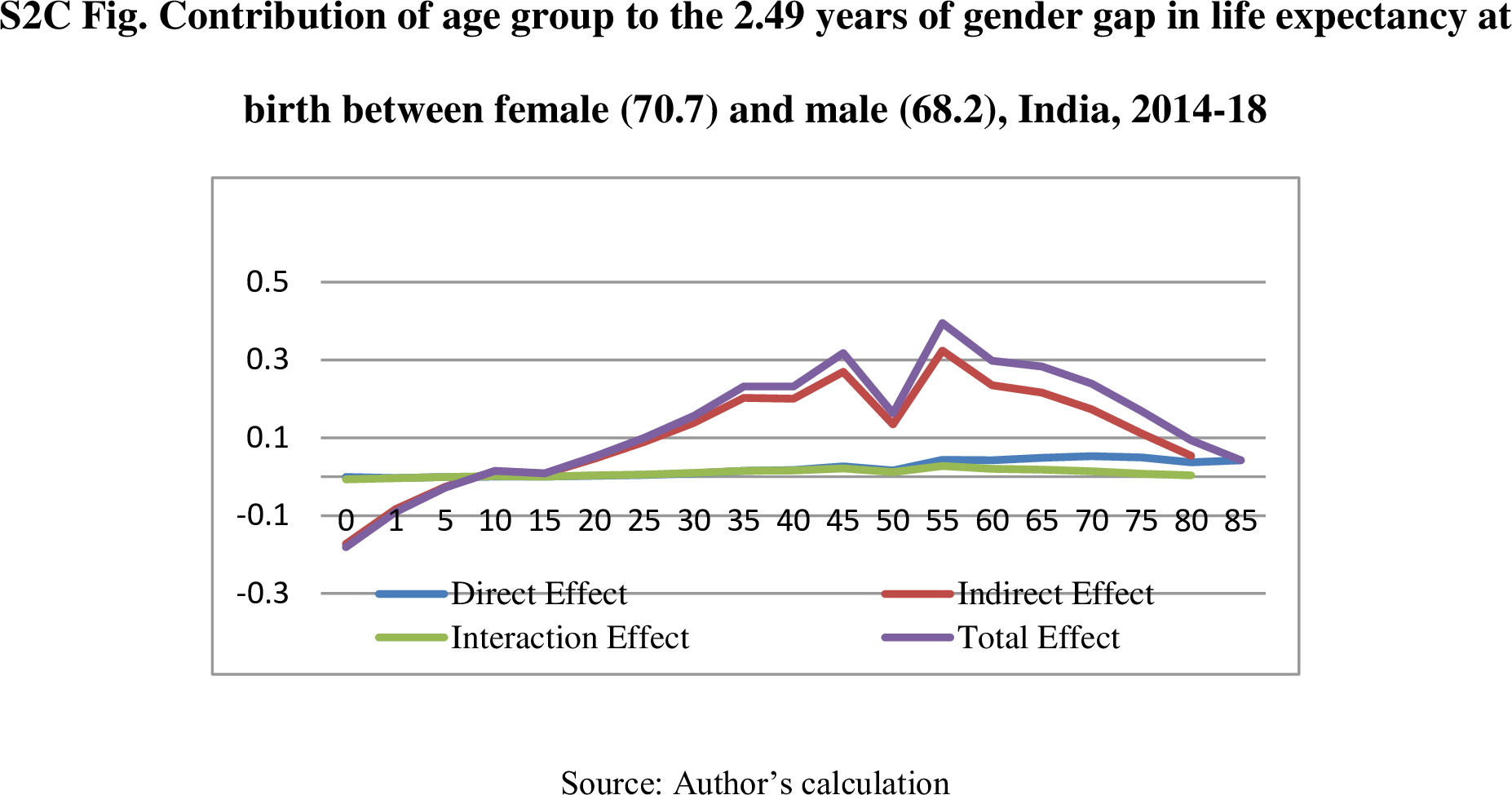

Supplement: S2 Fig — A. Contribution of age groups to the -1.69 years of gender gap in life expectancy at birth between female (48.80) and male (50.49), India, 1970–75. B. Contribution of age groups to the 0.06 years of gender gap in life expectancy at birth between female (55.33) and male (55.26), India, 1981–85. C. Contribution of age group to the 2.49 years of gender gap in life expectancy at birth between female (70.7) and male (68.2), India, 2014–18. Source: SRS and author’s calculation. (ZIP) [file pone.0260657.s002.zip › S2C_Fig.tif]

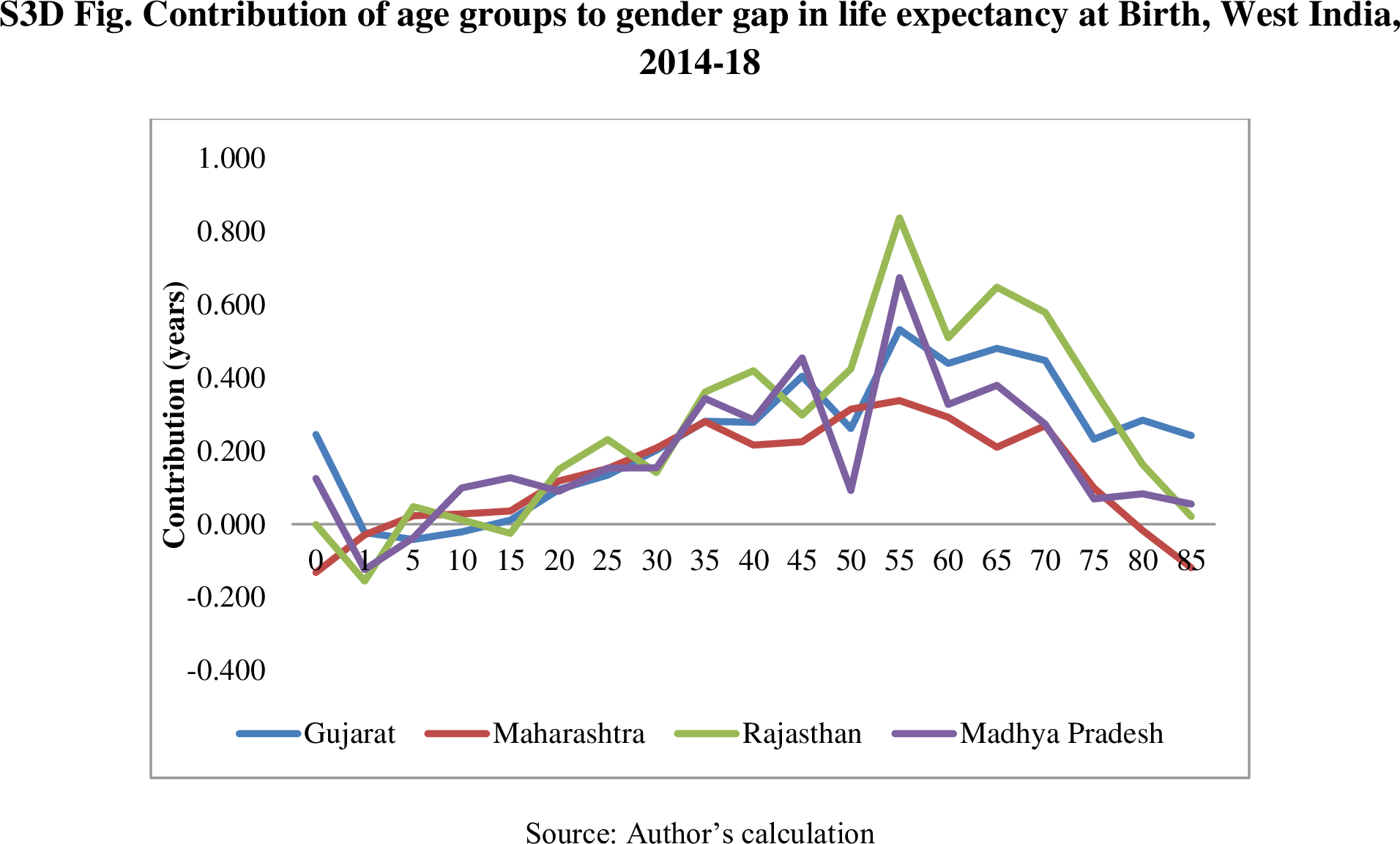

Supplement: S3 Fig — A. Contribution of age groups to gender gap in life expectancy at birth, South India, 2014–18. B. Contribution of age groups to gender gap in life expectancy at birth, North India, 2014–18. C. Contribution of age groups to gender gap in life expectancy at Birth, East India, 2014–18. D. Contribution of age groups to gender gap in life expectancy at Birth, West India, 2014–18. Source: SRS and author’s calculation. (ZIP) [file pone.0260657.s003.zip › S3D_Fig.tif]

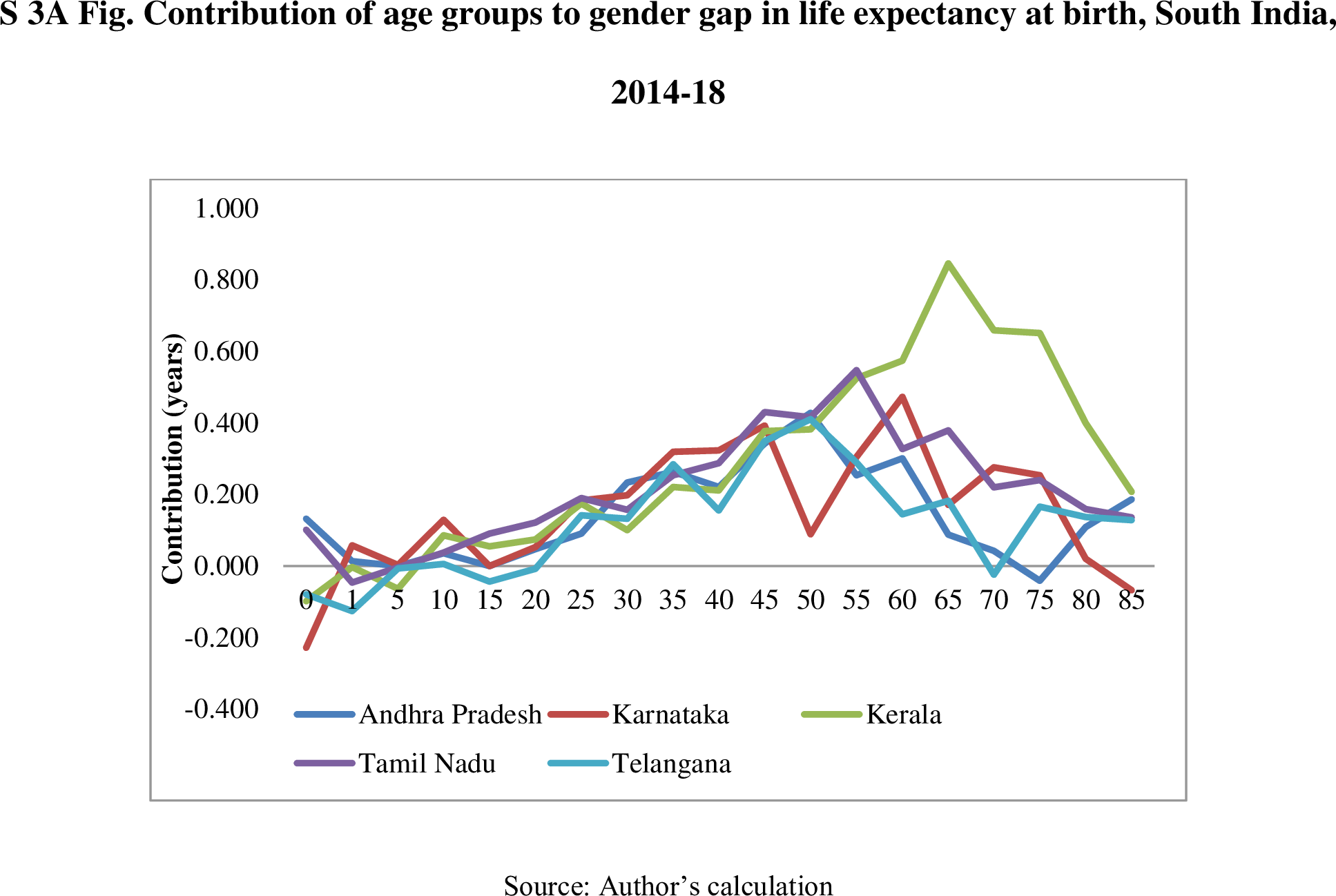

Supplement: S3 Fig — A. Contribution of age groups to gender gap in life expectancy at birth, South India, 2014–18. B. Contribution of age groups to gender gap in life expectancy at birth, North India, 2014–18. C. Contribution of age groups to gender gap in life expectancy at Birth, East India, 2014–18. D. Contribution of age groups to gender gap in life expectancy at Birth, West India, 2014–18. Source: SRS and author’s calculation. (ZIP) [file pone.0260657.s003.zip › S3A_Fig.tif]

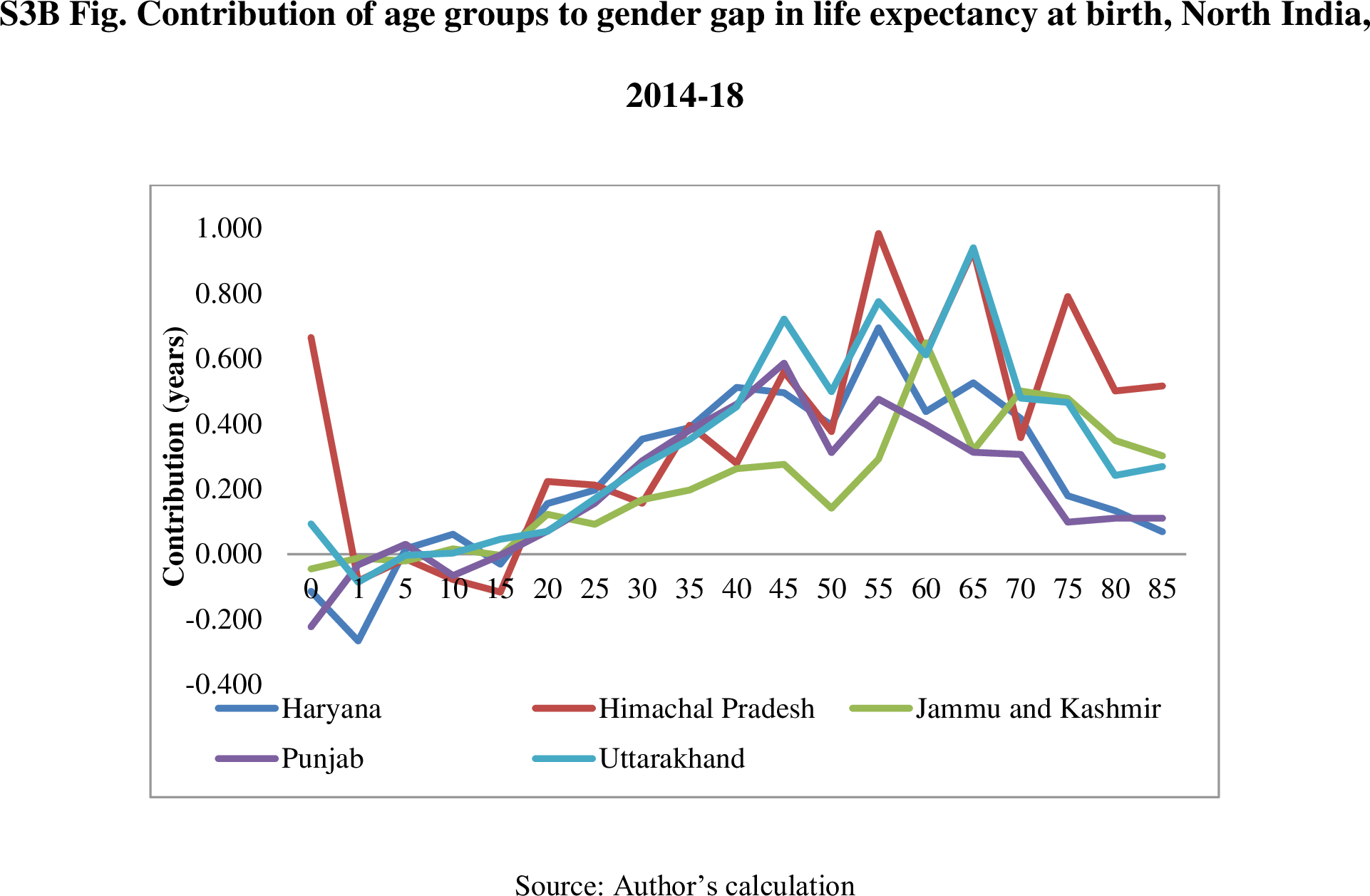

Supplement: S3 Fig — A. Contribution of age groups to gender gap in life expectancy at birth, South India, 2014–18. B. Contribution of age groups to gender gap in life expectancy at birth, North India, 2014–18. C. Contribution of age groups to gender gap in life expectancy at Birth, East India, 2014–18. D. Contribution of age groups to gender gap in life expectancy at Birth, West India, 2014–18. Source: SRS and author’s calculation. (ZIP) [file pone.0260657.s003.zip › S3B_Fig.tif]

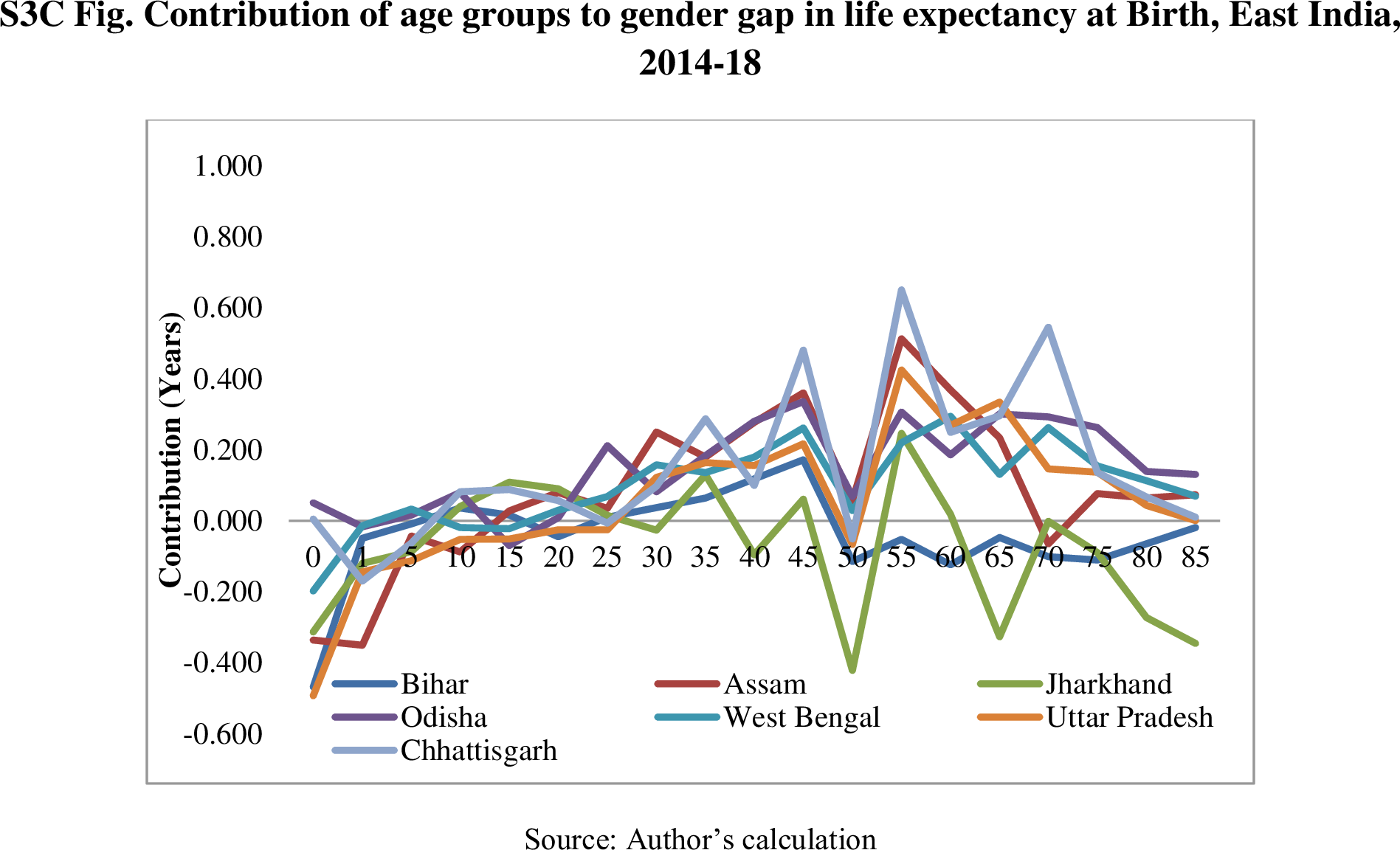

Supplement: S3 Fig — A. Contribution of age groups to gender gap in life expectancy at birth, South India, 2014–18. B. Contribution of age groups to gender gap in life expectancy at birth, North India, 2014–18. C. Contribution of age groups to gender gap in life expectancy at Birth, East India, 2014–18. D. Contribution of age groups to gender gap in life expectancy at Birth, West India, 2014–18. Source: SRS and author’s calculation. (ZIP) [file pone.0260657.s003.zip › S3C_Fig.tif]

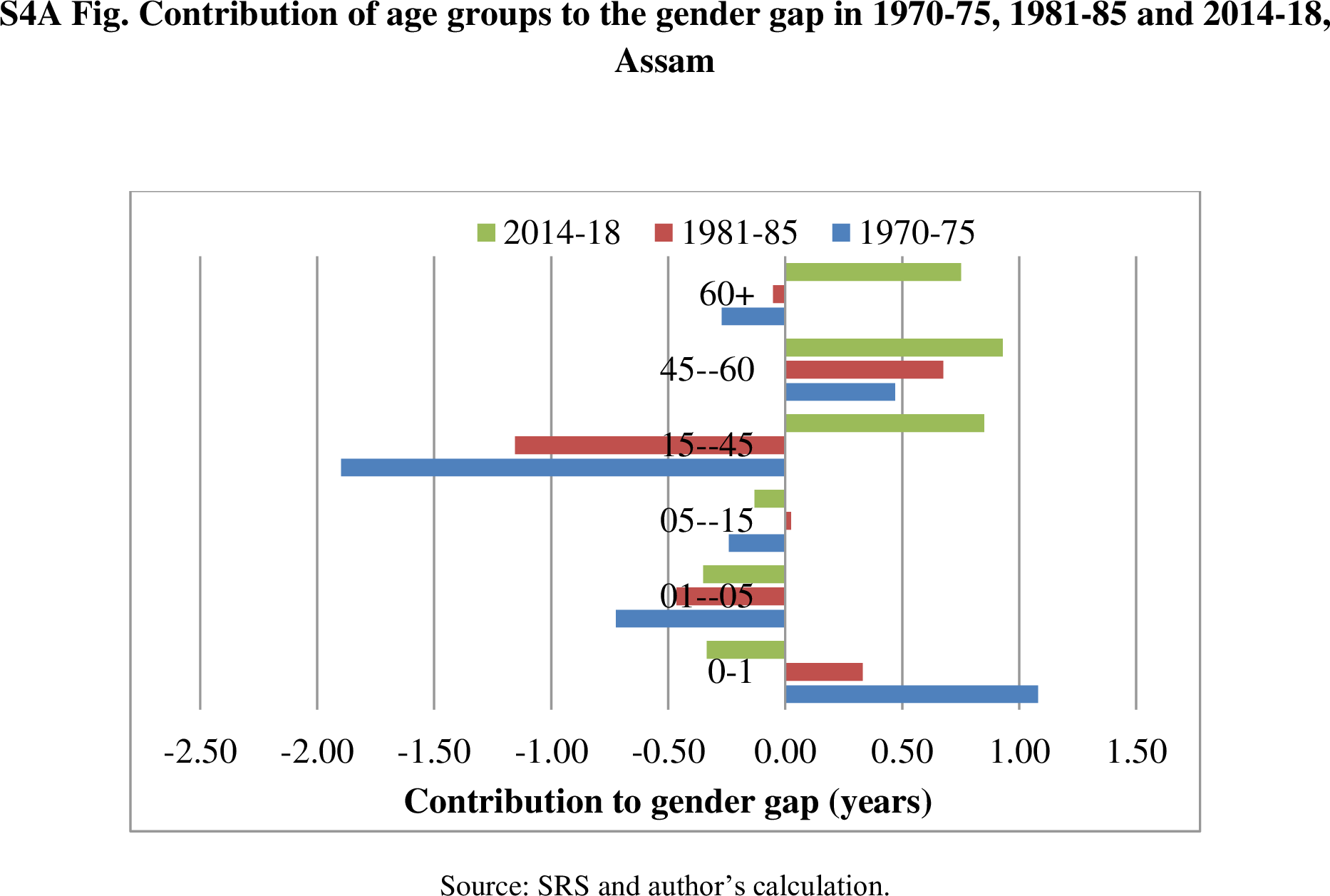

Supplement: S4 Fig — A. Contribution of age groups to gender gap in 1970–75, 1981–85 and 2014–18, Assam. B. Contribution of age groups to gender gap in 1981–85 and 2014–18, Bihar. C. Contribution of age groups to gender gap in 1970–75, 1981–85 and 2014–18, Madhya Pradesh. D. Contribution of age groups to gender gap in 1970–75, 1981–85 and 2014–18, Odisha. E. Contribution of age groups to gender gap in 1970–75, 1981–85 and 2014–18, Rajasthan. F. Contribution of age groups to gender gap in 1970–75, 1981–85 and 2014–18, Uttar Pradesh. Source: SRS and author’s calculation. (ZIP) [file pone.0260657.s004.zip › S4A_Fig.tif]

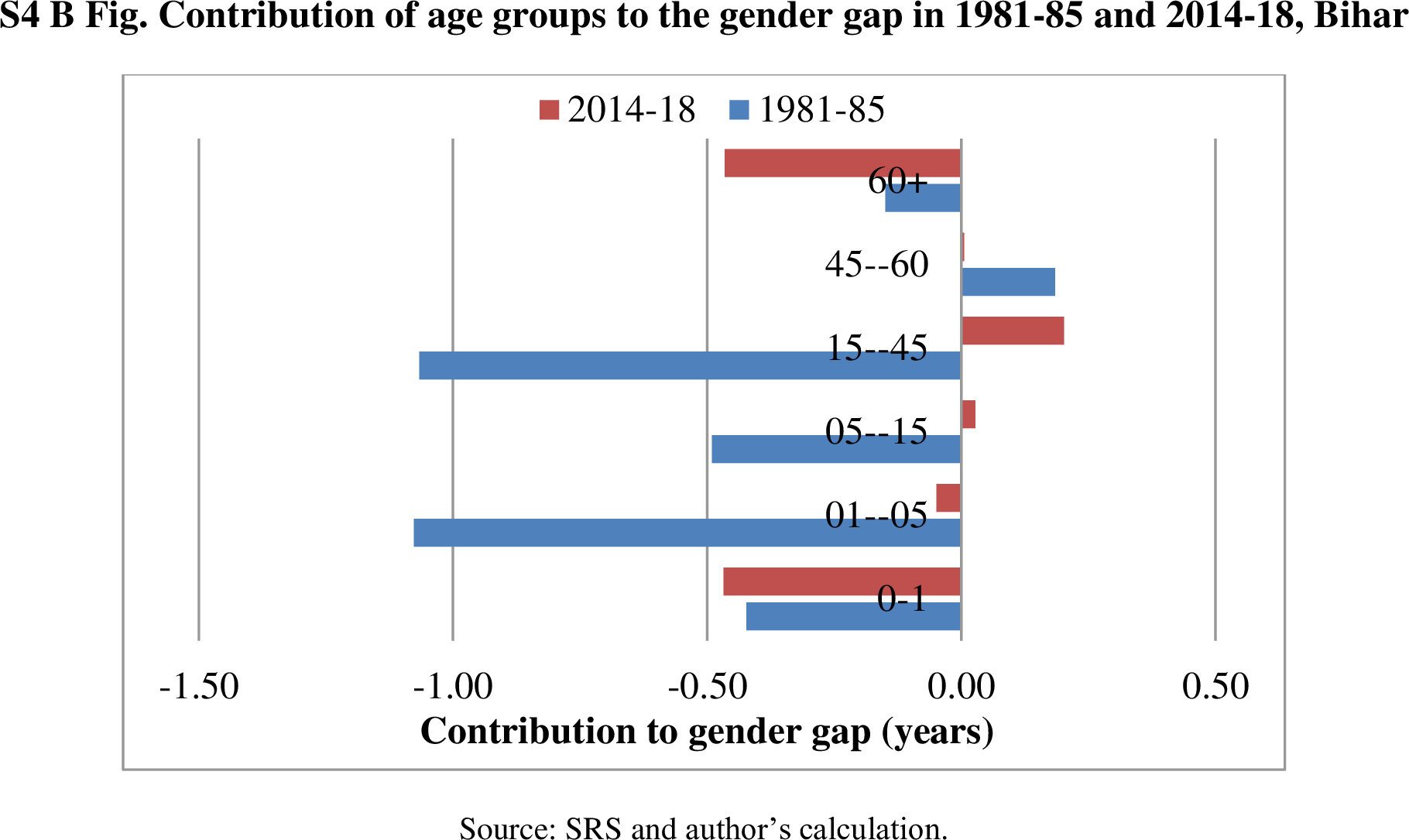

Supplement: S4 Fig — A. Contribution of age groups to gender gap in 1970–75, 1981–85 and 2014–18, Assam. B. Contribution of age groups to gender gap in 1981–85 and 2014–18, Bihar. C. Contribution of age groups to gender gap in 1970–75, 1981–85 and 2014–18, Madhya Pradesh. D. Contribution of age groups to gender gap in 1970–75, 1981–85 and 2014–18, Odisha. E. Contribution of age groups to gender gap in 1970–75, 1981–85 and 2014–18, Rajasthan. F. Contribution of age groups to gender gap in 1970–75, 1981–85 and 2014–18, Uttar Pradesh. Source: SRS and author’s calculation. (ZIP) [file pone.0260657.s004.zip › S4B_Fig.tif]

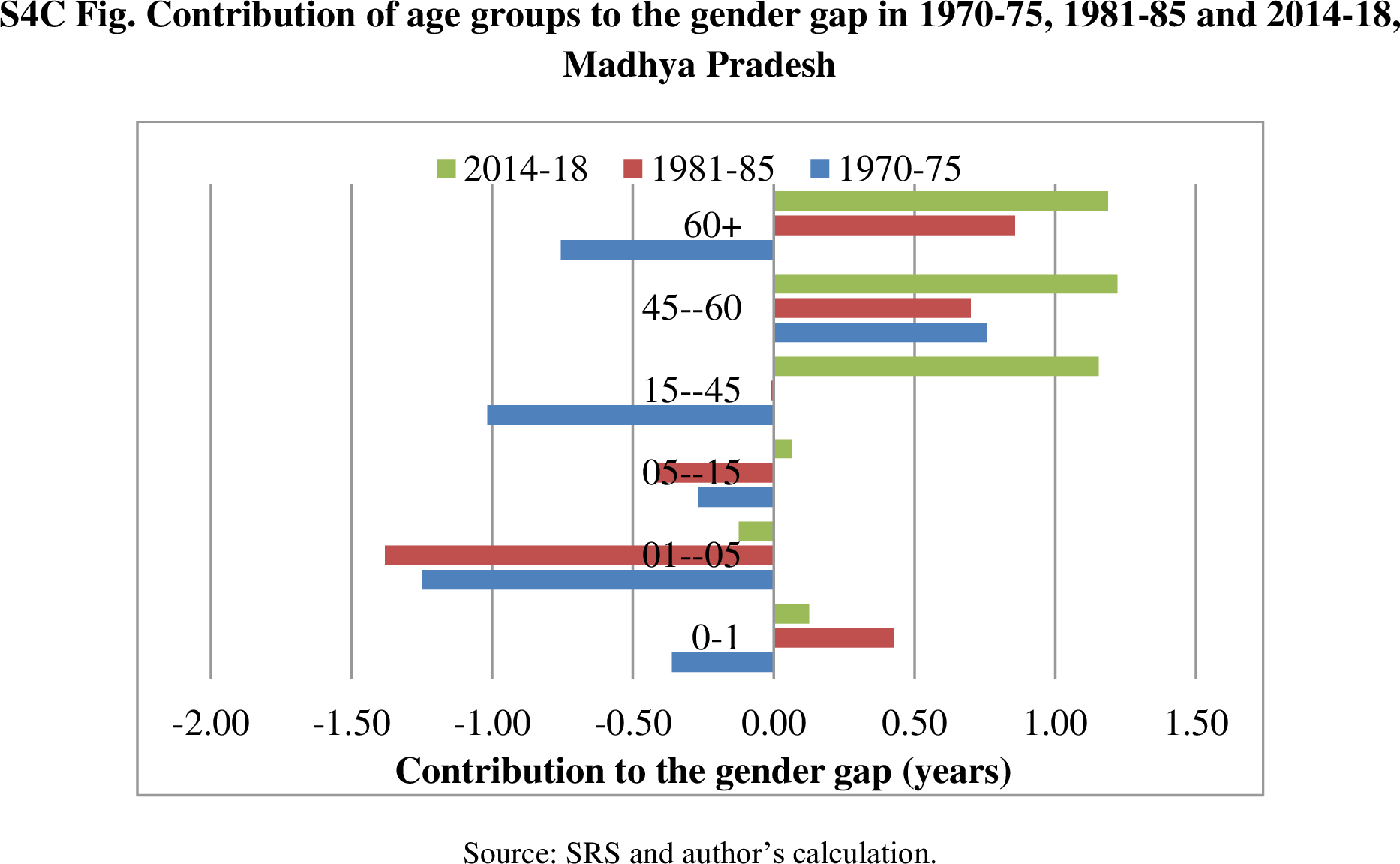

Supplement: S4 Fig — A. Contribution of age groups to gender gap in 1970–75, 1981–85 and 2014–18, Assam. B. Contribution of age groups to gender gap in 1981–85 and 2014–18, Bihar. C. Contribution of age groups to gender gap in 1970–75, 1981–85 and 2014–18, Madhya Pradesh. D. Contribution of age groups to gender gap in 1970–75, 1981–85 and 2014–18, Odisha. E. Contribution of age groups to gender gap in 1970–75, 1981–85 and 2014–18, Rajasthan. F. Contribution of age groups to gender gap in 1970–75, 1981–85 and 2014–18, Uttar Pradesh. Source: SRS and author’s calculation. (ZIP) [file pone.0260657.s004.zip › S4C_Fig.tif]

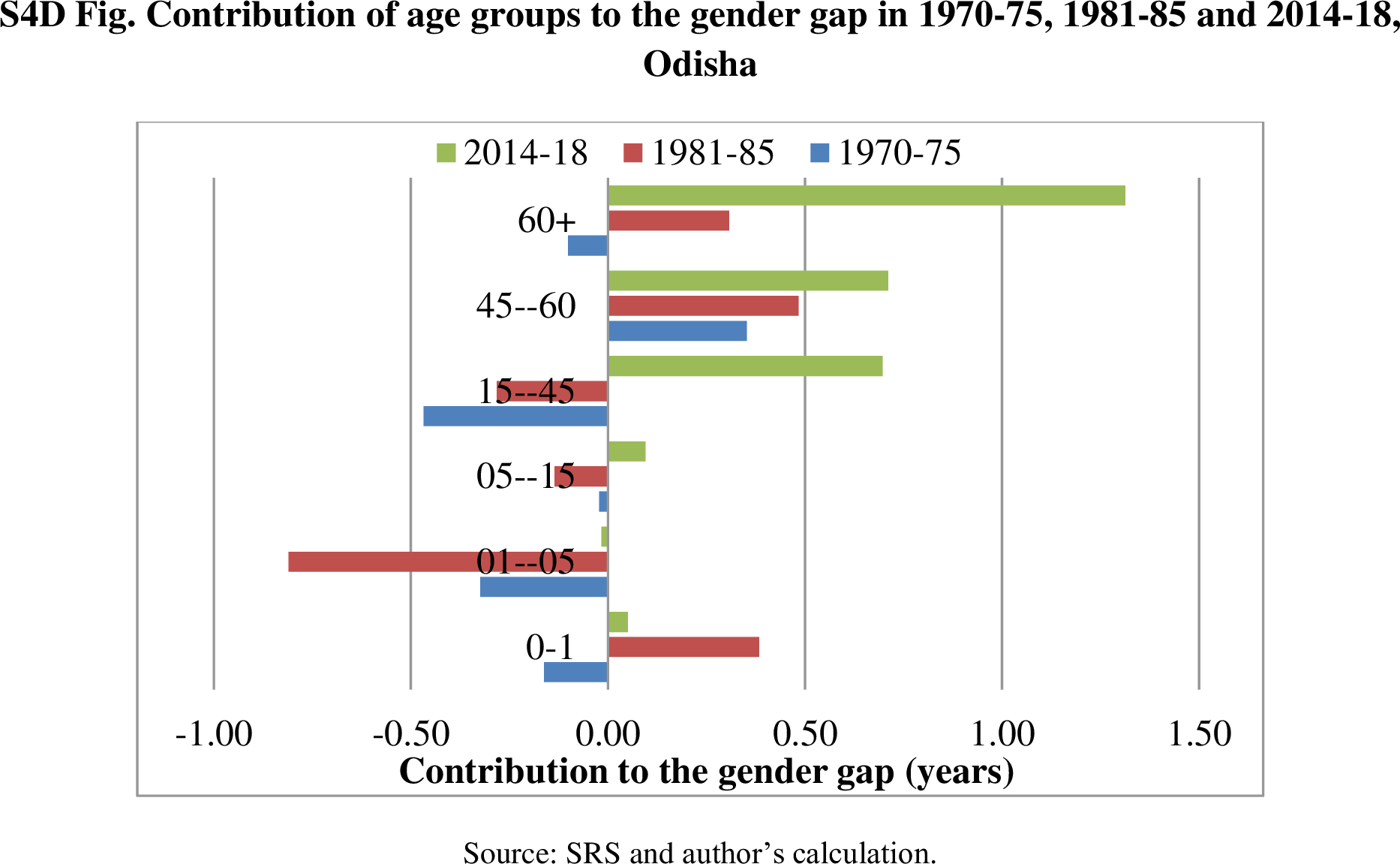

Supplement: S4 Fig — A. Contribution of age groups to gender gap in 1970–75, 1981–85 and 2014–18, Assam. B. Contribution of age groups to gender gap in 1981–85 and 2014–18, Bihar. C. Contribution of age groups to gender gap in 1970–75, 1981–85 and 2014–18, Madhya Pradesh. D. Contribution of age groups to gender gap in 1970–75, 1981–85 and 2014–18, Odisha. E. Contribution of age groups to gender gap in 1970–75, 1981–85 and 2014–18, Rajasthan. F. Contribution of age groups to gender gap in 1970–75, 1981–85 and 2014–18, Uttar Pradesh. Source: SRS and author’s calculation. (ZIP) [file pone.0260657.s004.zip › S4D_Fig.tif]

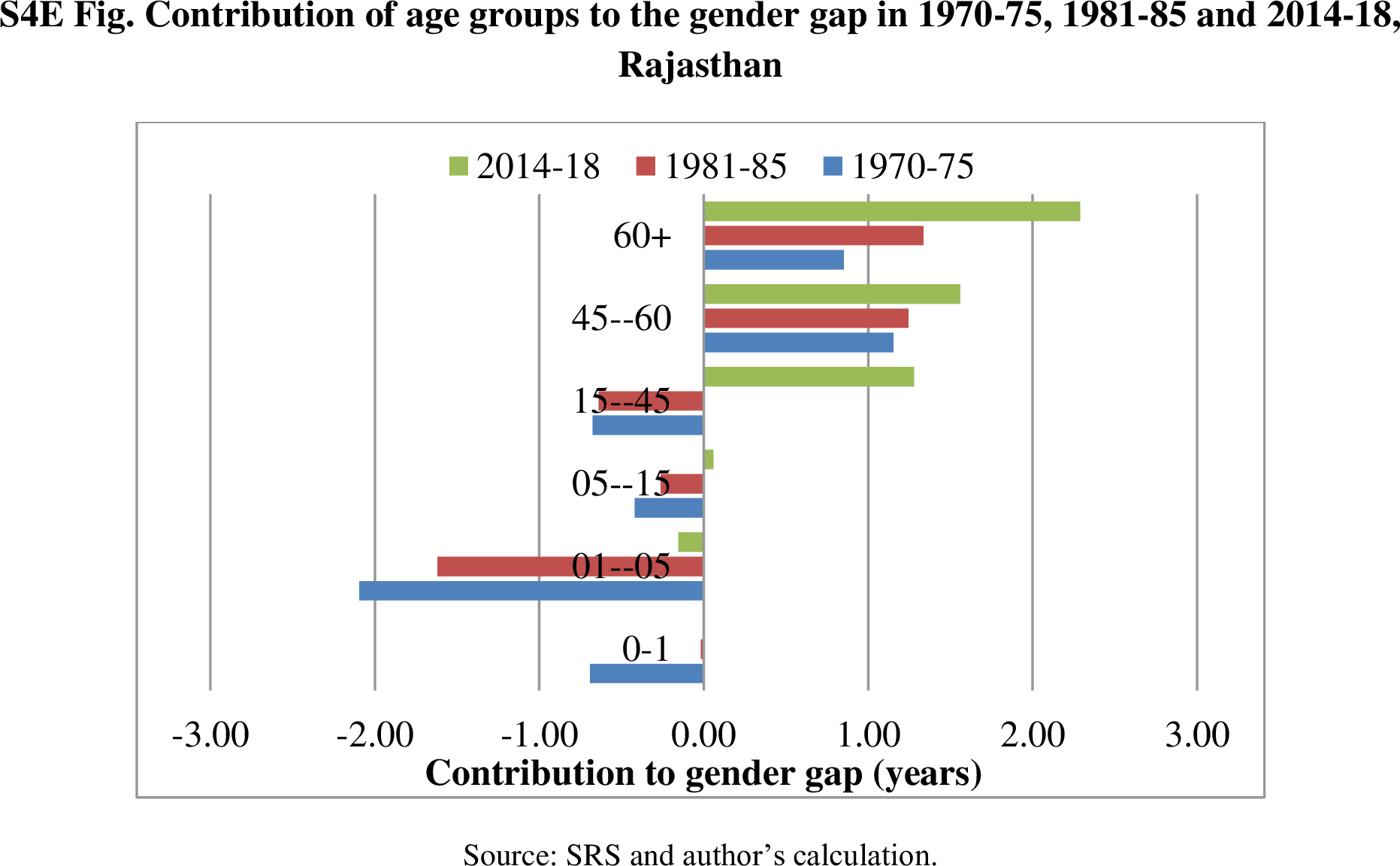

Supplement: S4 Fig — A. Contribution of age groups to gender gap in 1970–75, 1981–85 and 2014–18, Assam. B. Contribution of age groups to gender gap in 1981–85 and 2014–18, Bihar. C. Contribution of age groups to gender gap in 1970–75, 1981–85 and 2014–18, Madhya Pradesh. D. Contribution of age groups to gender gap in 1970–75, 1981–85 and 2014–18, Odisha. E. Contribution of age groups to gender gap in 1970–75, 1981–85 and 2014–18, Rajasthan. F. Contribution of age groups to gender gap in 1970–75, 1981–85 and 2014–18, Uttar Pradesh. Source: SRS and author’s calculation. (ZIP) [file pone.0260657.s004.zip › S4E_Fig.tif]

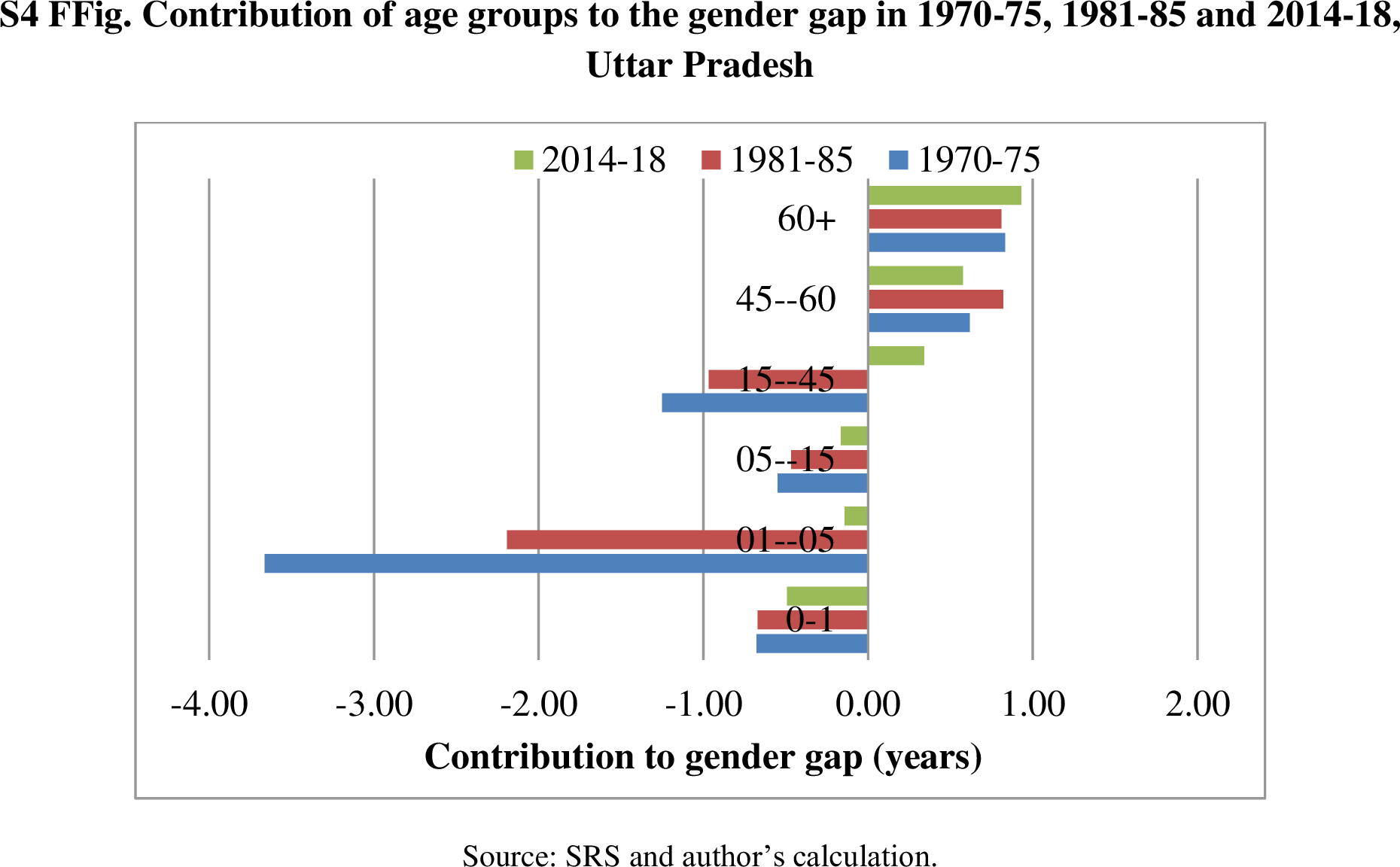

Supplement: S4 Fig — A. Contribution of age groups to gender gap in 1970–75, 1981–85 and 2014–18, Assam. B. Contribution of age groups to gender gap in 1981–85 and 2014–18, Bihar. C. Contribution of age groups to gender gap in 1970–75, 1981–85 and 2014–18, Madhya Pradesh. D. Contribution of age groups to gender gap in 1970–75, 1981–85 and 2014–18, Odisha. E. Contribution of age groups to gender gap in 1970–75, 1981–85 and 2014–18, Rajasthan. F. Contribution of age groups to gender gap in 1970–75, 1981–85 and 2014–18, Uttar Pradesh. Source: SRS and author’s calculation. (ZIP) [file pone.0260657.s004.zip › S4F_Fig.tif]
